# Use of Modeling to Inform Decision Making in North Carolina during the COVID-19 Pandemic: A Qualitative Study

**DOI:** 10.1177/23814683221116362

**Published:** 2022-07-29

**Authors:** Karl Johnson, Caitlin B. Biddell, Kristen Hassmiller Lich, Julie Swann, Paul Delamater, Maria Mayorga, Julie Ivy, Raymond L. Smith, Mehul D. Patel

**Affiliations:** Department of Health Policy and Management, Gillings School of Global Public Health, University of North Carolina at Chapel Hill, Chapel Hill, NC, USA; Department of Health Policy and Management, Gillings School of Global Public Health, University of North Carolina at Chapel Hill, Chapel Hill, NC, USA; Department of Health Policy and Management, Gillings School of Global Public Health, University of North Carolina at Chapel Hill, Chapel Hill, NC, USA; Department of Industrial and Systems Engineering, North Carolina State University, Atlanta, GA, USA; Department of Geography, University of North Carolina at Chapel Hill, Chapel Hill, NC, USA; Department of Industrial and Systems Engineering, North Carolina State University, Atlanta, GA, USA; Department of Industrial and Systems Engineering, North Carolina State University, Atlanta, GA, USA; Department of Engineering, College of Engineering and Technology, East Carolina University, Greenville, NC, USA; Department of Emergency Medicine, School of Medicine, University of North Carolina at Chapel Hill, Chapel Hill, NC, USA

**Keywords:** COVID-19, decision-making, modeling

## Abstract

**Background.** The COVID-19 pandemic has popularized computer-based decision-support models, which are commonly used to inform decision making amidst complexity. Understanding what organizational decision makers prefer from these models is needed to inform model development during this and future crises. **Methods.** We recruited and interviewed decision makers from North Carolina across 9 sectors to understand organizational decision-making processes during the first year of the COVID-19 pandemic (*N* = 44). For this study, we identified and analyzed a subset of responses from interviewees (*n* = 19) who reported using modeling to inform decision making. We used conventional content analysis to analyze themes from this convenience sample with respect to the source of models and their applications, the value of modeling and recommended applications, and hesitancies toward the use of models. **Results.** Models were used to compare trends in disease spread across localities, estimate the effects of social distancing policies, and allocate scarce resources, with some interviewees depending on multiple models. Decision makers desired more granular models, capable of projecting disease spread within subpopulations and estimating where local outbreaks could occur, and incorporating a broad set of outcomes, such as social well-being. Hesitancies to the use of modeling included doubts that models could reflect nuances of human behavior, concerns about the quality of data used in models, and the limited amount of modeling specific to the local context. **Conclusions.** Decision makers perceived modeling as valuable for informing organizational decisions yet described varied ability and willingness to use models for this purpose. These data present an opportunity to educate organizational decision makers on the merits of decision-support modeling and to inform modeling teams on how to build more responsive models that address the needs of organizational decision makers.

**Highlights:**

- Organizations from a diversity of sectors across North Carolina (including public health, education, business, government, religion, and public safety) have used decision-support modeling to inform decision making during COVID-19.
- Decision makers wish for models to project the spread of disease, especially at the local level (e.g., individual cities and counties), and to help estimate the outcomes of policies.
- Some organizational decision makers are hesitant to use modeling to inform their decisions, stemming from doubts that models could reflect nuances of human behavior, concerns about the accuracy and precision of data used in models, and the limited amount of modeling available at the local level.

## Introduction

The COVID-19 pandemic has challenged organizational decision makers to manage their organizations through a complex public health crisis.^1,2^ Throughout the ongoing crisis, many organizations have shifted the types of services offered, established structures for emergency decision making, and developed the capacity to track and act upon continuously updated COVID-19–related information.^3,4^ These changes have also been made within a broad and evolving landscape of decision making among other organizations, complicating any one decision maker’s ability to conceptualize the context around a decision or predict the likely impact of decision alternatives on their organization and the surrounding community.

In parallel, the pandemic has accelerated health sciences research and popularized many tools of health sciences researchers,^5,6^ including computer-based decision-support models (hereafter referred to as “models”).^7,8^ In the context of infectious disease, these models are built to simulate the future course of disease spread and subsequent outcomes under different policy scenarios and assumptions about human behavior and disease characteristics. These models have become well-known throughout the pandemic,^9^ especially those produced by major research institutions such as the Institute for Health Metrics and Evaluation at the University of Washington,^10^ Los Alamos National Laboratory,^11^ Northeastern University,^12^ Columbia University,^13^ and the Imperial College London.^14^ The results of such models have received notable attention among decision makers, defined as individuals whose job responsibilities include making decisions with a substantial impact on the structure of the organization or the individuals the organization serves. Throughout the pandemic, how decision makers across a range of organizational backgrounds (especially those outside public health or health care) have used these models is unclear. The perspectives of decision makers on the relevance and applicability of these models during COVID-19 are needed to inform model development during this and future crises.

In this qualitative study, we aimed to better understand the use of COVID-19 models for organization-level decision making. The data used for this study represent a convenience sample from a larger qualitative study on the context, processes, and inputs for organizational decision making among diverse sectors during the pandemic. Despite the limitations of this sampling frame, the conclusions of this study provide a foundation for further research on the understudied role of modeling in informing organizational decision-making.

## Methods

### Original Interview Recruitment and Design

Between October 2020 and February 2021, we conducted semi-structured interviews (45–60 min) with at least 3 decision makers from different organizations within each of the following 9 sectors: public health, public safety, county government, health care, business, transportation, religion, education, and community nonprofits. The characteristics of the individual organizations included in our original analysis are presented in Appendix 1. We defined organizations as consisting of any legally recognized entity, regardless of the number of members, volunteers, or employees. One member of the study team (K.J.) conducted all interviews using a secure web-based video-conferencing platform. To facilitate these interviews, 3 members of the study team (K.J., M.D.P., K.H.L.) developed a semi-structured interview guide and refined it over the first 3 interviews (Appendix 2). We asked interviewees about the decisions made or anticipated in response to COVID-19, along with the various factors and information that influenced or would better support their decisions.

To recruit decision makers, we used a snowball sampling approach,^15^ first by emailing individuals known by members of our research team (a team composed of health system engineers and public health scientists from 3 major North Carolina universities). We then asked interviewees for referrals to additional decision makers from other organizations who may provide a meaningful perspective on the questions asked, seeking diversity across organizations. This resulted in 120 potential interviewees being contacted and 44 interviewed (response rate: 37%). We determined the sample size based on reaching thematic saturation (defined as the point at which no new themes have emerged from the interviewees) across organizations within our established codebook domains while ensuring at least 3 interviews within each sector. The UNC Institutional Review Board determined this study was exempt from review.

### Analytical Approach

Given the open-ended structure of our interview guide and limited literature on decision making during COVID-19 at the time of analysis, we employed used conventional content analysis to derive themes from the interview transcripts.^16^ Using an inductive, iterative approach, we first outlined a preliminary codebook for each theme derived from the interview guide. Additional codes emerged as members of the research team analyzed a random sample of transcripts; new codes were added when data encountered did not fit into an existing code until no further codes were needed. Our completed codebook comprised the 6 major domains (organization background, decision inputs, decision context, decision processes, types of decisions made, and postdecision reflections) and minor codes within each major domain. When all interviews were completed and transcribed, the transcriptions and codebook were uploaded to MAXQDA 2018 qualitative analysis software (Verbi Software, 2009).^17^ Two independent members of the research team (C.B. and K.J) independently applied codes from the full codebook to each transcript before coming together to resolve discrepancies. Finally, 3 independent coders (C.B., K.J., H.H.) analyzed coded excerpts from all interviews within each minor code and, per conventional content analysis protocol, identified major and minor themes within each code with representative quotations. All coders were doctoral candidates with previous qualitative analysis experience. See Appendix 3 for more details on our methodological approach via the COnsolidated criteria for REporting Qualitative research Checklist (COREQ).^18^

### Analytic Sample Selection

For this analysis, we focus on a convenience sample of transcription segments concerned with the use of modeling as an input to inform decision making. Segments associated with additional codes are not included in this analysis but have been reported elsewhere.^19^ The content of modeling-specific segments consists of interviewee responses to the question of “what evidence/information/data is being used to make the decision” in response to COVID-19. The results of this analysis are derived from a subset of interviews in which the interviewer directly probed for modeling as a potential input for decision making. Given the nonacademic background of most of our interviewees, different types of simulation-based models (e.g., agent-based simulations, compartmental models, etc.) were not distinguished but treated as the general category “models.” To inform what was meant by the term “modeling” or “model,” the interviewer described and gave examples of models that have become popular during COVID-19 (i.e., the Institute for Health Metrics and Evaluation’s COVID-19 Projects model). If interviewees were still not aware of modeling after this explanation, no further modeling-related questions were asked (please see Appendix 4 for all included, de-identified modeling-specific responses to this question). There was a limited number of interviewee responses on modeling outside the context of pandemic response (e.g., revenue projections, student enrollment, airflow within buildings), but in keeping with the focus of our study, that content was not included in this analysis. Among those who did substantially discuss the role of modeling as an input for decision making during COVID-19, we identified key themes to help modelers appreciate current perceptions about modeling and opportunities to improve the use of modeling in decision-making.

## Results

Among the 44 decision makers interviewed in the larger analysis, nearly half (*n* = 19) discussed modeling as a decision input, and as a result, they were included in this analysis (Table 1). Interviewees from this subset came from the following sectors: county government (*n* = 2), health care (*n* = 3), local public health (*n* = 4), business (*n* = 1), public safety (*n* = 3), religion (*n* = 1), and education (*n* = 5). Most interviewees (*n* = 12) worked for organizations located in the central Piedmont region of the state that served a majority White community or constituent base (*n* = 13). For the individual organizational backgrounds of all those included in this analysis, please see Appendix 5.

**Table 1:** Interviewees Characteristics

Sector	No. of Interviewees	Organization Roles Included	Geographies Represented^a^	NC Regions Represented	Race/Ethnicities Represented^b^
Public safety	3	County sheriff, director of county emergency services, director of university emergency management	Metropolitan	Piedmont	Majority White, minority Black; majority White, minority Black and Asian
Health care	3	Systems engineer for private health system, president of healthcare association; director of student health services	Statewide, multi; metropolitan	Eastern; statewide	Majority white, minority Black and Asian; statewide association
Business	1	Director of public sector relations	Statewide	Statewide	Unknown
Public health	4	Director of local health department	Metropolitan	Piedmont	Majority White, minority Black; majority White, minority Black or Latino
Education	5	Senior vice provost; university president; county school board member (2); county school superintendent	Metropolitan	Piedmont; astern	Majority White, minority Black and Asian; minority White and Black; majority White, minority Black or Latino; minority White, Black, and Asian
Religion	1	Presbyterian minister	Metropolitan	Piedmont	Majority White
County government	2	County manager, assistant county manager	Nonmetropolitan	Western; eastern	Majority White

a Geography designations are based on data from the Office of Management and Budget’s metropolitan statistical area designations, which uses the county as the basic building block.

b Race/ethnicities were classified as “minority” if they constituted greater than 30% of the community/constituents but less than 50% and “majority” if they constituted greater than 50% of the community/constituents.

Reflections on the use of modeling among interviewees considered for this analysis were broadly categorized into the sources of models used in decision making, the applications for which decision makers used modeling, applications for modeling recommended by decision makers, and hesitancies toward model-informed decision making. The interviewees with commentary contributing to subthemes and representative quotations from among these interviewees are in Table 2.

**Table 2:** Major Interview Themes and Representative Quotes

Major Theme	Subtheme	Contributing Interviewees	Representative Quote
Source of models	Using multiple models	H3, PS3, PH3	“. . . we probably had about five different models that covered not only our state, and our local area and also compared that to the national level. . . . We weren’t going to just isolate or pick and choose certain things.” (Health care, H3)
	Use of national models	PH3, PH4, H5, G3	“Well, originally, we focused on a model out of the University of Washington. . . . That was one of the hospital CEOs we’re really looking at, and they lobbied Governor Cooper, for the stay at home order.” (Public health, PH3)
	Dependence on skilled model interpreter	PS4, E6, E4, R3, G3	“We weren’t out trying to vet the data or peer review it or any of those kinds of things. But our health director was taking the data she received from the CDC, she was taking the information she received from the North Carolina Department of Health and Human Services, she was taking the models that they were using to create the guides that they were giving. We took them to be trusted sources.” (Government, G3)
Applications of nodeling	Inform the allocation of scarce resources	PH3, B2, H3, E3	“So we’ve got operation models associated to the epidemiology, and then operation models help to indicate the supply chain logistics side to support that.” (Health care, H3)
	Project surge capacity	PS4, PH4	“The hospital surge capacity is where we start to get concerned. Any modeling that we see that shows that the hospital capacity is decreasing then we start to absolutely be concerned.” (Public safety, PS4)
	Project worst-case scenarios	G3, PS4, B2, H5	“We’re still going to do case investigations. We’re still going to do contact tracing. If anything, [modeling] tells us if there’s going to be that, ‘Oh, crap,’ moment.” (Public safety, PS4)
	Compare trends between localities	B2, PS3, H3	“[Modeling] allowed us to compare ourselves to other communities of similar size and density . . . [it] gave us a real gut check on how we were doing compared to other communities versus just the rest of North Carolina.” (Public safety, PS3)
	Communicate risk through model visualizations	H3, H5, PH1	“We all learn from . . . visual cues. [Modeling is] not abstract. I mean, it is abstract, but it’s not abstract words, it’s that hard stuff you can hang your hat on. . . . That was probably the most valuable thing . . . it’s that visualization and just putting it in front of people’s faces. Because otherwise, it’s all words.” (Health care, H5)
Recommended applications for modeling	Predict disease spread within subpopulations	PS4, H5	“If we could know, like what cluster we are looking at next, what group of people could we potentially be looking at next. We can start tailoring our message and get out to those trusted leaders to say, ‘Look, you are not immune from this. This could very well affect you too.’” (Public safety, PS4)
	Inform safe reopening strategies	PH4, H1	“I would love to see the simulations, and this is what I think everybody would love to see, what happens if we get to a space where we fully reopen and we take the limits off of some of the occupancy/capacity. . . . Would it be enough to do the masking and the hand-washing? . . . So maybe with the simulation and modeling, it would take into account if you had immunity amongst this percentage of the population because to me, maybe that would give us some goals in our vaccination planning and programs. ‘Well, if we get to this, we stand a better chance of this outcome.’” (Public health, PH4)
Challenges with and hesitancies toward modeling	Sensitivity to model assumptions	H5, E1, PH5	“It’s just finding out or trying to put any signs we’re using or modeling we’re using in the proper context, knowing its limitations and so forth because everyone’s using it as like here’s the answer. No, that’s a possible answer certainly, but it may not be the answer, and what are the other possibilities of how accurate are these models. What’s your point of failure? In what ranges of circumstances are they useful and outside of those ranges do they lose that? I’m still a bit puzzled on the whole thing.” (Education, E1)
	Difficulties with incorporating the nuances of human behavior	PH5, PS7, E5	“So those models could be helpful. But how do you factor behavior in those models? Assuming you can, and I do think you can, I mean, I do work with some of these models as well, and you can account for that kind of by different parameters in the model of that estimate how close people are in contact with each other, how many people are wearing masks, stuff like that. Compliance is the issue, I think.” (Education, E5)
	Inappropriate data used to develop models	H5, PH5, E1	“I think modeling would help. . . . I think we need to be a lot more transparent with the data to comfort the people.” (Education, E1)
	Inability of state-level models to translate to local contexts	PH5	“The other problem was a lot of the models are at the very best are state level models. They’re not local. And because we looked pretty different from the rest of the state in terms of numbers and those sorts, they wanted to see something from here. . . . The modeling for me would be very helpful if there was a focus on what’s going on here . . . it doesn’t help me to look at the state and see that their numbers are going one way and mine are going the other.” (Public health, PH5)

### Source of Models

Interviewees in our analytic sample followed models at multiple scales, including local, state, and/or national levels, from a variety of sources. Some organizations built their own internal models, including private health systems, large public health departments, and university settings with modeling-trained scientists; however, most relied on results generated by external modeling groups. In the absence of local modeling resources, a few decision makers relied on national models, especially those from the Institute for Health Metrics and Evaluation at the University of Washington, to inform initial decision making around shutdowns and safety protocols. Three decision makers commented on the use of multiple models to inform decision making, even the desire to collect as many models as they could to include diverse perspectives. Justifications for this approach included the recognition that one model could not provide all the answers (they did not want to “isolate or pick and choose certain things”; health care 3), that it was important to consider models operating at different scales, and to compare external models to internal, in-house models to confirm that decision making at the local level made sense given state- or national-level trends. This aligns with a general wariness, as expressed by most interviewees, toward making a decision that would draw negative attention to their locality or organization if their local model was wrong.

Having someone in the organization who was trained to interpret models, point to key takeaways, and situate findings within the broader literature was expressed as a facilitator to using models to inform decision making among several different organizations. In the absence of this internal knowledge, a few interviewees noted using only models that had been approved by their local health director as a “trusted source” (government 1) who could discern which models were credible.

### Applications of Modeling

Four different decision makers used models to inform the allocation of scarce resources and critical care employees (e.g., where to stock personal protective equipment). Identifying different possible disease trajectories facilitated the estimation of needed resources in the future and activated the right plan to secure those resources. There was a clear linkage among these organizations between patterns in epidemiological models and decision making based on their own operational or logistical models. Relatedly, models that estimated the impact of disease spread on hospital surge capacity were commonly used for decision making. Such models gave decision makers a sense of when demand for health care–related services would be the most intense. More broadly, several interviewees commented on the use of modeling to estimate the worst-case scenarios and when the worst may occur (the “oh, crap moment,” public safety 1) and then to compare these scenarios to their available resources. When the estimated risk surpassed what they were capable of responding to, some decision makers discussed how more conservative actions were then taken.

A few interviewees described the usefulness of modeling in terms that suggested its ability to confirm (provide a “sanity check” or “gut check”) their personal intuition about how the pandemic was progressing or the effectiveness of their policies. In other instances, decision makers used modeling to monitor disease spread in “other communities of similar size and density” (public safety 3) to estimate what could be expected in their own jurisdiction. Comparisons occurred not just within North Carolina but also across other states, especially those that had been hardest hit by the Pandemic early on. Throughout these comparisons, the implicit suggestion was that what had occurred or may be occurring elsewhere may inform what could happen to their own locality, prompting the need to prepare for the coming wave. Other respondents emphasized how valuable modeling was for tracking the spread of disease within small geographic areas, especially those corresponding with vulnerable communities that could be reached through targeted communication, testing, and contact-tracing efforts.

In several instances, decision makers emphasized the value of model visualizations to clarify the impact of policies on disease spread for multiple audiences. Visualizations helped to make the abstract complexity of models more concrete conceptually. Compared with merely discussing individual data points, model visualizations provided “hard stuff you can hang your hat on” (health care 1). This was especially the case for clarifying how bad the spread of disease could become. These visuals were also educational. One public health official (public health 1) described working with a government epidemiologist as they presented graphically how different policies may shift the “curve” of total infections over time in a way that was both intuitive and easily actionable. By making scenarios and potential outcomes more concrete, model visualizations informed their decision making amid the complexities of COVID-19.

### Recommended Applications for Modeling

Multiple decision makers proposed applications for which modeling would be useful. For example, a handful of individuals emphasized a desire for models to show disease spread within subpopulations, including by race/ethnicity, to understand and predict how groups experiencing a higher burden of disease shifted over time. There was a desire to know “what cluster we are looking at next” (public safety 1) to prepare targeted messaging. A local health director desired modeling to help directly inform when and how it would be safe to return to in-person meetings. This included the desire to show the outcomes of social distancing policies and specifically to determine what minimum number of restrictions need to be in place to ensure a safe reopening. They also proposed modeling to inform communication campaigns for vaccination rollout, with a particular interest in identifying target coverage levels (“well if we get to this, we stand a better chance of this outcome,” public safety 1). A director of student health services at a public university (health care 1) similarly desired modeling to inform testing policies if schools were to return in-person. Another interviewee expressed a desire for modeling the downstream consequences of the pandemic beyond typical health-related outcomes, particularly assessing developmental outcomes for kids who have been in school virtually.

### Challenges with and Hesitancies toward the Use of Models

Many interviewees expressed generally positive comments about modeling, such that models “kind of give insight” (health care 3), “played a key role” (public health 1), and helped them avoid “making the wrong decision” (education 1). However, even among those who gave favorable commentary, interviewees expressed various potential concerns about modeling in general and the specific models on offer during the pandemic. This included the recognition that models can be highly uncertain and sensitive to differing assumptions, which may lead to both inconsistent results across different models and inaccurate results when comparing projected estimates with what actually occurred. Inconsistent results were considered especially problematic, given the challenge of providing consistent messaging about COVID-19–related topics to the public. Especially among public health officials, communicating modeling-informed findings to the community was considered difficult for this reason, given the scrutiny with which their constituents followed public data on the spread of disease. Additional concerns included doubts that models could effectively incorporate the nuances of human behavior, particularly compliance with official mask usage guidelines. Among such interviewees, there was an implicit assumption that models must rigidly mirror official guidelines (e.g., everyone wearing masks). Other decision makers questioned the quality of data used in the models, acknowledging that “models are only as good as the comfort level you have with the data going into it and the assumptions being made” (health care 1). This critique acknowledged that models could become an effective tool for decision making if quality data were used and if these data were transparently presented. Another concern raised was that models did not capture the nuances of local communities; decision makers identified unique characteristics of their own communities, which made them substantively different from the state at large. One local health director (public health 2) commented on the inability of state-level model results to provide useful information for individual counties. In the absence of modeling-informed decision making, local decision makers described following COVID-19–related data daily and retrospectively examining where their locality may be with respect to a state-level prediction or the projected impact of prior intervention.

## Discussion

Among COVID-19 decision makers interviewed for our study, about half commented on the use of models to inform decision making (including reasons for not using them). Among this convenience sample of interviewees, modeling was viewed as valuable for understanding the trajectory of the pandemic within their communities and informing organizational decisions, such as opening facilities or allocating resources. Visualizations were valuable for understanding and communicating model outcomes. Notable points of hesitancy toward the use of modeling results included the limited number of models focusing on local contexts, doubts about how well models could incorporate the nuances of human behavior, and the perceived high sensitivity of modeling results to changes in modeling assumptions.

Our study results emphasize the need to modernize and standardize the data infrastructure in the United States to support these model development and calibration needs. Both accurate modeling at the local level and modeling the impact of policy among subpopulations (especially vulnerable communities) demand access to granular data, which was difficult to obtain during COVID-19 (these include mobility data or data on mask usage, neither of which are easily accessible by age and race or ethnicity).^20,21^ In the absence of internal modeling capacity and data collection locally, local decision makers may have to rely on externally built models that may not consider important features of the local context.^22^ To advance the uptake of modeling across all levels of decision making, it is important to develop further modeling capacity at the local level, to train decision makers in how to translate the findings of models at larger scales to their own context, and to appreciate the variety of modeling objectives (e.g., to include both projection as well as learning about the relative impact of intervention alternatives in the presence of uncertainty).

Given the hesitancies raised in our study along with support from prior studies on the effective implementation of decision-support models during COVID-19 and best modeling practices before the pandemic began,^23,24^ additional guidance is needed to help local decision makers become better consumers of modeling. This guidance should focus on building capacity to understand the objectives, scope, and policy context of model conceptualization and different model outcomes, such as predictions of new infections or deaths over time, intervention rankings, or impact of differences. Assisting decision makers to be effective communicators of model-informed decisions is also crucial. Guidance should also be given to help decision makers understand measures of uncertainty associated with model structure and estimates and the forms of modeling bias.^25^ This is especially so given the public resistance following from what are perceived to be inaccurate model predictions in which model uncertainty was not transparently reported.^26^ Inspired by findings from our study and the experiences of other modeling teams during the pandemic, additional research should be conducted on the strategies by which findings produced from simulation models can be transparently and accessibly presented to both the public and decision makers.^27^

Given the convenience sample from which our results are derived, we encourage further studies to assess the ways in which diverse sectors use modeling to inform decision making. The findings of our study identify 3 major categories in which further research can be conducted: the sources of models, the diverse applications of modeling (desired and realized), and challenges with model-informed decision making.

## Limitations

This study has several limitations that should be noted when considering the practical implications of our results. Because the interviewees included in this study of the use of modeling are a subset of those interviewed on decision making during the first year of the COVID-19 pandemic, there are potential sources of bias. First, although we were able to reach thematic saturation among those included in our original sample, we cannot confirm thematic saturation among the subsample included in this analysis. In addition, as compared with all of those interviewed, those included in this study had a higher percentage representation from the health care, public health, and education sectors. Furthermore, it is possible that those not included in this analysis had salient perspectives on modeling, but the questions included within our interview guide did not probe for them. While this may be due to limitations of our interview guide, it may also suggest a generally limited awareness of modeling as a tool among decision makers and how it differs from, for example, merely presenting data trends on a COVID-19 dashboard. Future studies should consistently probe for the level of awareness organizational decision makers have about modeling. Regardless of the cause, we cannot know whether these excluded comments (a form of missing data) would have reinforced or contradicted commentary among those included in this analysis. Our findings suggest the need for future studies that examine the perspectives of decision makers who used and did not use modeling. Moreover, our study of 19 interviewees represents diverse organizational perspectives spanning 7 sectors, and there were between 1 and 5 interviewees per sector. To improve the actionability of findings, future research should focus on the use of modeling within specific sectors in contrast to our goal of capturing cross-sector perspectives. Specific attention should be given to sectors that were underrepresented in this analysis (e.g., transportation, nonprofit organizations, religious organizations), given the novelty with which these sectors may be implementing the use of modeling for decision making. Relatedly, the snowball sampling strategy used to contact potential interviewees may have biased our sample to include those with a greater familiarity with modeling than the general population, given the prior relationships these individuals had with members of our research team. However, only 4 of those interviewed had such relationships (Appendix 5). Finally, as discussed earlier, we treated modeling as a general category throughout our interviews and did not distinguish between the different types of modeling methods. While this approach captured a broad range of modeling-related commentary, it limited our ability to address specific examples or use cases of modeling. We encourage future studies to interview decision makers on specific modeling forms to fill this gap, potentially soliciting feedback on specific examples of different types of models given the high level of unfamiliarity among those whom we interviewed.

## Conclusion

Through a convenience sample of semi-structured interviews with decision makers across multiple sectors in North Carolina, this study found mixed appreciation for the presence and use of decision-support models to inform decision making during the first year of the COVID-19 pandemic. Whereas many decision makers appreciated the ability to use models to understand trends in disease spread and the effectiveness of future policies, the lack of modeling at the local level and concerns about the perceived sensitivity of modeling results to changing assumptions limited widespread uptake. The findings of this study should be used to inform the design and dissemination of future decision-support models (for the COVID-19 pandemic and future crises) and to train decision makers on how to become better consumers of decision-support models that are relevant to decisions within their organization.

## Supplemental Material

sj-docx-1-mpp-10.1177_23814683221116362 – Supplemental material for Use of Modeling to Inform Decision Making in North Carolina during the COVID-19 Pandemic: A Qualitative Study Click here for additional data file. Supplemental material, sj-docx-1-mpp-10.1177_23814683221116362 for Use of Modeling to Inform Decision Making in North Carolina during the COVID-19 Pandemic: A Qualitative Study by Karl Johnson, Caitlin B. Biddell, Kristen Hassmiller Lich, Julie Swann, Paul Delamater, Maria Mayorga, Julie Ivy, Raymond L. Smith and Mehul D. Patel in MDM Policy & Practice

sj-docx-2-mpp-10.1177_23814683221116362 – Supplemental material for Use of Modeling to Inform Decision Making in North Carolina during the COVID-19 Pandemic: A Qualitative Study Click here for additional data file. Supplemental material, sj-docx-2-mpp-10.1177_23814683221116362 for Use of Modeling to Inform Decision Making in North Carolina during the COVID-19 Pandemic: A Qualitative Study by Karl Johnson, Caitlin B. Biddell, Kristen Hassmiller Lich, Julie Swann, Paul Delamater, Maria Mayorga, Julie Ivy, Raymond L. Smith and Mehul D. Patel in MDM Policy & Practice

sj-docx-4-mpp-10.1177_23814683221116362 – Supplemental material for Use of Modeling to Inform Decision Making in North Carolina during the COVID-19 Pandemic: A Qualitative Study Click here for additional data file. Supplemental material, sj-docx-4-mpp-10.1177_23814683221116362 for Use of Modeling to Inform Decision Making in North Carolina during the COVID-19 Pandemic: A Qualitative Study by Karl Johnson, Caitlin B. Biddell, Kristen Hassmiller Lich, Julie Swann, Paul Delamater, Maria Mayorga, Julie Ivy, Raymond L. Smith and Mehul D. Patel in MDM Policy & Practice

sj-docx-5-mpp-10.1177_23814683221116362 – Supplemental material for Use of Modeling to Inform Decision Making in North Carolina during the COVID-19 Pandemic: A Qualitative Study Click here for additional data file. Supplemental material, sj-docx-5-mpp-10.1177_23814683221116362 for Use of Modeling to Inform Decision Making in North Carolina during the COVID-19 Pandemic: A Qualitative Study by Karl Johnson, Caitlin B. Biddell, Kristen Hassmiller Lich, Julie Swann, Paul Delamater, Maria Mayorga, Julie Ivy, Raymond L. Smith and Mehul D. Patel in MDM Policy & Practice

sj-pdf-3-mpp-10.1177_23814683221116362 – Supplemental material for Use of Modeling to Inform Decision Making in North Carolina during the COVID-19 Pandemic: A Qualitative Study Click here for additional data file. Supplemental material, sj-pdf-3-mpp-10.1177_23814683221116362 for Use of Modeling to Inform Decision Making in North Carolina during the COVID-19 Pandemic: A Qualitative Study by Karl Johnson, Caitlin B. Biddell, Kristen Hassmiller Lich, Julie Swann, Paul Delamater, Maria Mayorga, Julie Ivy, Raymond L. Smith and Mehul D. Patel in MDM Policy & Practice

## References

[1] Comfort LK Kapucu N Ko K Menoni S Siciliano M. Crisis decision-making on a global scale: Transition from cognition to collective action under threat of COVID-19. Public Admin Rev. 2020;80(4):616–22. 10.1111/puar.13252 PMC7300963 32836462

[2] Berger L Berger N Bosetti V , et al. Uncertainty and decision-making during a crisis: How to make policy decisions in the COVID-19 context? University of Chicago, Becker Friedman Institute for Economics Working Paper. 2020;2020-95.

[3] Seetharaman P. Business models shifts: impact of COVID-19. Int J Inform Manag. 2020;54:102173. 10.1016/j.ijinfomgt.2020.102173 PMC7323683 32834338

[4] Obrenovic B Du J Godinic D Tsoy D Khan MA Jakhongirov I. Sustaining enterprise operations and productivity during the COVID-19 pandemic: “Enterprise Effectiveness and Sustainability Model.” Sustainability. 2020;12(15):5981.

[5] Tufekci Z. Don’t believe the COVID-19 models. Atlantic. April 2, 2020. Available from: www.theatlantic.com/technology/archive/2020/04/coronavirus-models-arent-supposed-be-right/609271/. Accessed December 28, 2021.

[6] Hsu J. Here’s how computer models simulate the future spread of new coronavirus. Scientific American. February 13, 2020. Available from: www.scientificamerican.com/article/heres-how-computer-models-simulate-the-future-spread-of-new-coronavirus/. Accessed December 28, 2021.

[7] Callaway E Ledford H Viglione G Watson T Witze A. COVID and 2020: an extraordinary year for science. Nature. 2020;588(7839):550–2. 10.1038/d41586-020-03437-4 33318685

[8] Kinsella CM Santos PD Postigo-Hidalgo I , et al. Preparedness needs research: how fundamental science and international collaboration accelerated the response to COVID-19. PLoS Pathogens. 2020;16(10):e1008902. 10.1371/journal.ppat.1008902 PMC7546461 33035262

[9] Adam D. Special report: the simulations driving the world’s response to COVID-19. Nature. 2020;580(7802):316–9. 10.1038/d41586-020-01003-6 32242115

[10] IHME COVID-19 Forecasting Team. Modeling COVID-19 scenarios for the United States. Nat Med. 2021;27:94–105. 33097835 10.1038/s41591-020-1132-9 PMC7806509

[11] Castro L Fairchild G Michaud I Osthus D. COFFEE: COVID-19 forecasts using fast evaluations and estimation. arXiv preprint. 2021;arXiv:2110.01546.

[12] Vespignani A Tian H Dye C , et al. Modelling covid-19. Nat Rev Physics. 2020;2(6):279–81. 10.1038/s42254-020-0178-4 PMC7201389 33728401

[13] Branas CC Rundle A Pei S , et al. Flattening the curve before it flattens us: hospital critical care capacity limits and mortality from novel coronavirus (SARS-CoV2) cases in US counties. medRxiv. January 1, 2020.

[14] Wise J. Covid-19: risk of second wave is very real, say researchers. BMJ. 2020;369:m2294. 10.1136/bmj.m2294 32518177

[15] Naderifar M Goli H Ghaljaie F. Snowball sampling: a purposeful method of sampling in qualitative research. Strides in Development of Medical Education. 2017;14(3).

[16] Hsieh HF Shannon SE. Three approaches to qualitative content analysis. Qual Health Res. 2005;15(9):1277–88. 10.1177/1049732305276687 16204405

[17] VERBI Software. MAXQDA 2020 [computer software]. Berlin: VERBI Software; 2019. Available from: maxqda.com

[18] Tong A Sainsbury P Craig J. Consolidated criteria for reporting qualitative research (COREQ): a 32-item checklist for interviews and focus groups. Int J Qual Health Care. 2007;19(6):349–57. 10.1093/intqhc/mzm042 17872937

[19] Biddell CB Johnson K Patel MD , et al. Cross-sector decision landscape in response to COVID-19: a qualitative analysis of North Carolina decision-makers. medRxiv. January 1, 2022. 10.3389/fpubh.2022.906602 PMC9424900 36052008

[20] Badr HS Gardner LM. Limitations of using mobile phone data to model COVID-19 transmission in the USA. Lancet Infect Dis. 2021;21(5):e113. 10.1016/S1473-3099(20)30861-6 PMC7837090 33152270

[21] Gandhi RT Lynch JB Del Rio C. Mild or moderate Covid-19. N Engl J Med. 2020;383(18):1757–66. 10.1056/NEJMcp2009249 32329974

[22] Jewell NP Lewnard JA Jewell BL. Predictive mathematical models of the COVID-19 pandemic: underlying principles and value of projections. JAMA. 2020;323(19):1893–4. 10.1001/jama.2020.6585 32297897

[23] James LP Salomon JA Buckee CO Menzies NA. The use and misuse of mathematical modeling for infectious disease policymaking: lessons for the COVID-19 pandemic. Med Decis Making. 2021;41(4):379–85. 10.1177/0272989X21990391 PMC7862917 33535889

[24] Roberts M Russell LB Paltiel AD Chambers M McEwan P Krahn M. Conceptualizing a model: a report of the ISPOR-SMDM modeling good research practices task force–2. Med Decis Making. 2012;32(5):678–89. 10.1177/0272989X12454941 22990083

[25] Siegenfeld AF Taleb NN Bar-Yam Y. Opinion: what models can and cannot tell us about COVID-19. Proc Natl Acad Sci U S A. 2020;117(28):16092–5. 10.1073/pnas.2011542117 PMC7368306 32581126

[26] Eker S. Validity and usefulness of COVID-19 models. Humanities and Social Sciences Communications. 2020;7(1):1–5.

[27] Nosyk B Weiner J Krebs E , et al. Dissemination science to advance the use of simulation modeling: our obligation moving forward. Med Decis Making. 2020;40(6):718–21. 10.1177/0272989X20945308 PMC7484337 32755285

