# Supplementary material for: Use of Modeling to Inform Decision Making in North Carolina during the COVID-19 Pandemic: A Qualitative Study
Source: MDM Policy Pract. 2022 Jul 29;7(2):23814683221116362. doi: 10.1177/23814683221116362 (PMC9340948; doi:10.1177/23814683221116362)
Supplement: sj-docx-1-mpp-10.1177_23814683221116362 – Supplemental material for Use of Modeling to Inform Decision Making in North Carolina during the COVID-19 Pandemic: A Qualitative Study [file sj-docx-1-mpp-10.1177_23814683221116362.docx]

**Appendix 1 -- Characteristics of Participating Organizations from Original Interviewee Sample**

| **Sector** | **Total Interviewees** | **Organization Types Represented** | **Geographies Represented** | **NC Regions Represented** | **Race/Ethnicities Represented** |
| --- | --- | --- | --- | --- | --- |
| Business | 4 | Real estate, Retail shop, Coffee shop, Software company | State-wide, Counties in metro areas of fewer than 250,000 population | State-wide, Eastern | Majority Black, Minority White Town; Majority white town; unknown |
| Non-profit organization | 3 | Recreation & youth programming, charity food distribution | Counties in metro areas of 1 million population or more, Multi-regional | Piedmont, Eastern | Majority Hispanic, Minority White, Asian, and Middle-Eastern; unknown |
| County Government | 4 | County Management, County Social Services | Counties in metro areas of 250,000 to 1 million population; Completely rural or less than 2,500 urban population, not adjacent to a metro area; Urban population of 20,000 or more, adjacent to a metro area | Western, Eastern, Piedmont | Majority White, Minority Black; Majority White; Majority White, Minority Black and Hispanic |
| Healthcare | 5 | Healthcare association/society, Private health system, University student health | Regional, state-wide | State-wide, Eastern |  |
| Public Health | 5 | Local health departments (LHDs) | Counties in metro areas of 250,000 to 1 million population, Counties in metro areas of 1 million population or more | Western, Piedmont | Majority White, Minority Black or Latino; Majority White, Minority Black |
| Public Safety | 7 | County Emergency Services/Management, County Sheriff's Office | Counties in metro areas of 1 million population or more, Counties in metro areas of fewer than 250,000 population, Counties in metro areas of 250,000 to 1 million population, Urban population of 20,000 or more, adjacent to a metro area | Piedmont, Eastern, Western | Majority White, Minority Black; Majority White |
| Religious Organization | 6 | Christian Churches (Evangelical, Methodist, Presbyterian, Catholic) | Counties in metro areas of fewer than 250,000 population, Counties in metro areas of 1 million population or more, Counties in metro areas of 250,000 to 1 million population | Eastern, Piedmont | Majority Black; Minority Black and Minority White; Majority White; Majority Hispanic |
| Education | 7 | Universities, Community college, Private & public grade schools, school board | Counties in metro areas of 1 million population or more, Counties in metro areas of fewer than 250,000 population, Counties in metro areas of 250,000 to 1 million population | Piedmont, Eastern | Majority White, Minority Black and Asian; Minority White and Black; Majority White, Minority Black or Latino; Minority White, Black, and Asian; Majority White; Unknown |
| Transportation | 3 | City Transportation, State DOT | Counties in metro areas of 250,000 to 1 million population; State-wide | Western, Piedmont, State-wide | Majority White, Minority Black; State-wide |
