# Supplementary material for: Use of Modeling to Inform Decision Making in North Carolina during the COVID-19 Pandemic: A Qualitative Study
Source: MDM Policy Pract. 2022 Jul 29;7(2):23814683221116362. doi: 10.1177/23814683221116362 (PMC9340948; doi:10.1177/23814683221116362)
Supplement: sj-docx-2-mpp-10.1177_23814683221116362 – Supplemental material for Use of Modeling to Inform Decision Making in North Carolina during the COVID-19 Pandemic: A Qualitative Study [file sj-docx-2-mpp-10.1177_23814683221116362.docx]

Appendix 2: Semi-structured Interview Guide

**Verbal Consent**

The purpose of this research study is to determine key policies and interventions that are currently being considered for the COVID-19 response and planning at the state and local levels. Our multidisciplinary team from UNC, NC State, and ECU is investigating how best to minimize the impact of COVID-19 on the citizens of North Carolina. You are being asked to take part in this research study because of your [ORGANIZATION ROLE]. Sharing your insights and perspectives with us will ensure the right questions are being asked and results are produced in a way that will be most useful to state-level and local decision makers.

We will not ask any questions or obtain any information related to your personal health or other private or sensitive topics. We are only interested in your organization’s response and planning for the current COVID-19 pandemic. The primary risk to you is the inadvertent disclosure of your participation in this research. To minimize this potential loss of confidentiality, we will store your name, contact information, and interview responses on a secure server with access restricted to only team members.  The audio will also be recorded, so we can refer back to it to make sure we did not miss anything. Only team members will have access to these recordings. We will delete everything once no longer needed. We will only share aggregate results in presentations and publications, and no result or response will be attributed to an individual person.

Participating in this research study is completely voluntary. You can choose not to be in this research study. You can also say yes now and change your mind later. During the interview, you can choose not to answer any question you do not wish to answer. You can also choose to stop the interview at any time.

Do you have any questions for me about this research or your potential participation?

Would you like to participate in the interview for this study, and do you agree to be recorded?

*[After consent is given]*

**Pre-COVID Questions**

*Intro:* *we’d first like to ask a few general questions about the background of your organization, without yet considering the COVID-19 pandemic.*

- Could you please describe, in your opinion, your organization’s mission?
  - What main services do you provide the community? (if not obvious)
  - Which communities do you serve?
- What is your role in the organization?
  - What are your primary responsibilities?
  - Who are the people you report to?
  - Who are the people who report to you?

**General COVID Questions**

*Let’s now transition to the rest of our questions, those which deal specifically with the COVID-19 Pandemic. We’d first like to ask a few questions about how your organization approached the Pandemic.*

- How does your organization talk about the risks from COVID-19?
  - Risks regarding the potential spread of infection within your organization
  - Risks regarding the potential spread of infection from people in your organization to those your organization interacts with.
- Can you describe how you see your organization’s role regarding the pandemic during?
- Can you describe your organization’s role regarding reducing risk from the pandemic?
  - Within the organization?
  - Within the wider community?
- How do your organization see its role in developing safety protocols?
  - Responsibility?
- How does your organization see its role in ensuring testing and tracing?
- Can you describe the thoughts you have on people’s individual responsibility to reduce risk?
  - How do the responsibilities of your organization interface with people’s individual responsibility to reduce risk?
  - How do your personal perspectives on risk and risk reduction compare to your organization’s perspectives?
- Are there ways in which you believe your personal perspective on risk and risk reduction differs from the perspective of your organization?
  - Can you tell me more about that?

**Early (March-April) COVID Questions**

*Segue: Next, we’d like to ask a few questions about decisions and decision-making in your organization during the context of COVID-19.*

*We’d like for you to walk us through some specific decisions your organization has had to make during the COVID-19 pandemic and how those decisions were made.*

- In your organization, what key macro decisions did you make as an initial response to COVID-19 back in March-April? (Please consider decisions that had a big impact on on the organization or those whom the organization effects.)
- Safety measures?
- Staffing changes?
- New services?
- Opening-up / closing-down protocols?
- Anything regarding social distancing, mask usage, public relations messaging
- Can you walk me through how (one of these key decisions) were made?
  - What happened first?
  - Who was involved?
  - What happened next?
  - What, if anything, do you wish had been different about that decision?
- What alternatives were you choosing among?
  - What stakeholder considerations were you weighing when deciding between options?
- Public interest
- Public opinion
- Investors/funders
- Bad/good press
- Customer/consumers/participants
- Organization staff
- What were the most important outcomes that are informing the decision?
- How certain were you of the future outcome of the decision? How have you sought to gain more certainty?
- What parties/stakeholders were involved in those decisions?
  - What unique roles do each contribute to the decision-making process?
- What evidence/information/data was used to make the decision?
  - What was your greatest need for evidence/information/data?
  - What was the best way in which information/data or other inputs could be presented to inform this decision?
  - Do you think it’s the responsibility of the organization or a separate authority to provide this information?
- Do you wish you could have formalized the decision-making process during that time?
  - Can you tell me more about that?
- What was the most difficult thing about organizational decision-making during the first few months of the Pandemic? How could these difficulties be overcome, or how were they overcome for your organization?
  - Too much information to process?
  - Information keeps changing?
  - Unclear who the proper authorities are?

**Current/Future COVID Questions**

*Now we’d like to shift our focus to the current and future context of the Pandemic.*

- How have discussions regarding your organization’s role changed since last spring?
  - How has your org’s perception of risk changed since last spring?
- How is your organization talking about what might happen in the next several months?
- In your organization, what key macro decisions are you currently considering, say over the next 3-6 months? (Please consider decisions that had a big impact on the organization or those whom the organization effects.)
- Can you walk me through how (one of these key decisions) is being made?
  - What happened first?
  - Who was involved?
  - What happened next?
  - What, if anything, do you wish had been different about that decision?
- What alternatives are you choosing among?
- What stakeholder considerations are you weighing when deciding between options?
  - Public interest
  - Public opinion
  - Investors/funders
  - Bad/good press
  - Customer/consumers/participants
  - Organization staff
- What are the most important outcomes that are informing the decision?
- How certain are you of the future outcome of the decision? How are you seeking to gain more certainty?
- What parties/stakeholders are involved in those decisions?
  - What unique roles do each contribute to the decision-making process?
- What evidence/information/data is being used to make the decision?
  - What is your greatest need for evidence/information/data?
  - What is the best way in which information/data or other inputs could be presented to inform this decision?
  - Do you think it’s the responsibility of the organization or a separate authority to provide this information?
- What is the most difficult thing about organizational decision-making right now? How could these difficulties be overcome, or how are they being overcome for your organization?
  - Too much information to process?
  - Information keeps changing?
  - Unclear who the proper authorities are?
- Do you wish you could have formalized the decision-making process?
  - Can you tell me more about that?

*Finally, we’d like to discuss how the decision-making process in your organization may have changed during COVID-19.*

- Before COVID-19, what was the general decision making process like?
  - How were decisions made?
  - How hierarchical was the decision-making process?
  - What was a typical timeline for decision-making?
  - Who made the final decision?
- How has the general decision-making process changed during the COVID-19 pandemic, if at all?
  - More hierarchical? New staff roles?
  - Task forces developed?
  - Faster pace than normal?
- How have these inputs/factors you consider in the decision-making process changed during the COVID-19 pandemic, if at all?
  - New authorities? New inputs?
  - New staff dedicated to processing inputs?
  - Have these inputs changed during the course of the pandemic?

*Closing: We greatly appreciate your input… We’re aiming to learn from a wide range of perspectives and collect a diversity of opinions.*

- Thinking back on your answers, is there anyone else in your organization or related organizations you know of whom we should talk to get a more complete picture or a differing perspective on this topic?
