# Supplementary material for: Use of Modeling to Inform Decision Making in North Carolina during the COVID-19 Pandemic: A Qualitative Study
Source: MDM Policy Pract. 2022 Jul 29;7(2):23814683221116362. doi: 10.1177/23814683221116362 (PMC9340948; doi:10.1177/23814683221116362)
Supplement: sj-docx-4-mpp-10.1177_23814683221116362 – Supplemental material for Use of Modeling to Inform Decision Making in North Carolina during the COVID-19 Pandemic: A Qualitative Study [file sj-docx-4-mpp-10.1177_23814683221116362.docx]

**Appendix 4 -- All Included Interviewee Responses (De-identified)**

| **Interviewee** | **Modeling-related Responses** |
| --- | --- |
| PS4 | Interviewer:  ...if that kind of modeling has been used at all throughout the course of your   decision making during the pandemic? And whether or not the answer to that, looking forward, how do   you think it could be helpful in helping you guys make decisions?  Respondent:  So we have both our own internal with our epidemiologists of what the numbers are looking like. We   receive UNIVERSITY’S COVID modeling on a weekly basis, if I'm not mistaken. And we also received some from   the international innovative emergency management in LOCATION as well, we receive that modeling, use   spies. By way of what the pandemic is going to do, it doesn't necessarily change what our response is   going to be. We're still going to do case investigations. We're still going to do contact tracing. If   anything, it tells us if there's going to be that, Oh, crap, moment. We were relying very heavily on the   university medical system to let us know what their current status is? How many beds they have   available? And if they're going to be activating into a surge capacity or not?  I believe that the hospital surge capacity is where we start to get concerned. At any modeling that we   see that shows that the hospital capacity is decreasing then we start to absolutely be concerned. But it   doesn't change what our response is going to be. It's more of which plan are we going to have to   activate to be able to deal with the surge capacity? Does that make sense?  Yeah. And maybe just to keep unpacking this, do you think if you had more control over these models or   more direction on what they were analyzing, what would you use them for? If anything, what kind of   questions do you think could be helpful to know? What hypothetically could happen under different   scenarios, different policy interventions, or a few months out, what's it going to look like?  So I think the... we saw in LOCATION a very interesting transition in who had COVID at what point. And we   work very... emergency management works very hard in the prevention realm and so does public   health. They're very much about preventing public health diseases. We saw the writing on the wall in   the very beginning that it was going to be minority populations that were going to be affected the   hardest. And that deals with health disparities, access to care, all of those things are issues that the   pandemic has only exacerbated. So we saw it go from a black African-American community to a Latinx   and Hispanic community. And now it's gone to a white community. And so what would be...? The   modeling would be really interesting to see what was happening historically? What was happening from   a sociological perspective? As to why it was going through these groups and why is it just now? What   started as a rich white person disease, right?  Because you had to have the ability to be able to travel, came back to affect the black African-American   community then affects the Hispanic and Latinx community, because they are essential workers as   classified by the pandemic, they are the essential workers. And is now affecting the white community. Is   it because we haven't...? We as white people have not been affected to date? And so we must be good   to go. We found out that Hispanic and Latinx community believed that they had some type of immunity   to COVID-19 and couldn't catch it. And we very much found out that that was not the case.   But if we could know, like what cluster we are looking at next, what group of people could we   potentially be looking at next. We can start tailoring our message and get out to those trusted leaders to   say, "Look, you are not immune from this. This could very well affect you too." This is the group of   people that you need to look at now. And I don't know that that type of modeling exists. I know the   number modeling exist. But who's it going to be next? And let us get in front of it that way. |
| PS3 | Interviewer:  Maybe in that same spirit, as I had mentioned earlier, we're working with some folks from NC State that   do up a lot of infectious disease modeling, so trying to simulate, project the future of the pandemic   under different policy scenarios and determining what's going to be more or less effective. I'm curious,   at one level, whether any of those kinds of models were used in any decision making process or, if not,   maybe it seems like it is, how could they be useful going forward?  Respondent:  Well, here's an inside story that is really interesting. We have three different model sets that we would   watch. One is our own internal modeling system. We have a data scientist that I brought onboard three   and a half years ago. It just turns out, I'm going to call him a kid because he's much younger than I am,   but this person is a whizz. He's a state graduate, digs what we're doing, and his models were really, and   are, really, really good. We would compare that, his models, as far as what we're seeing on ground and based off of prior week/prior month what we can expect. We would use Duke's modeling. Then also out of North Carolina   Emergency Management they had a vendor, BUSINESS, that puts out a daily model as well. We would constantly compare their models to ours and where we were actually. I don't know that we based decisions off of those two models. We used those to fact-check ourselves and to make sure there was some type of sanity check with the protective actions that we were putting in place here locally.  Interviewer: Yeah. I want to bite at that for a second. The last question I have for this modeling work is what do you  think the most useful things ... Maybe not the two other models but the internal one, was able to do for   you all? Insofar as informing decision making.  Respondent:  I think it allowed us to compare ourselves to other communities of similar size and density. What some   of the models do and others don't, you can look at certain communities and their numbers might look  horrible or their numbers might look really good, based off of total population, but when you look at the   population density of NAME County compared to, say, NAME County, maybe even in NAME County, or any other   community [inaudible 00:42:14] County, whatever, our modeling gave us a real gut check on how we   were doing compared to other communities versus just the rest of North Carolina. We would specifically look at folks that had similar populations, folks that had similar population densities, and then we could track where we were compared to them because we know they're going through the same thing that we're going through but if our restrictions were in place longer and we're doing better than they are, we know our protective measures are working. I think that internal modeling or that internal piece for us let us do that. |
| B2 | Respondent:  They didn't have a sense of how do I get all this organized in a way that I can quickly understand what it means, right? But we're not even talking in the analytics. We're just saying, "How do we get this data in a place where I can consume it? And then I can start to think about it." Maybe the exception to that was, places like XXX Clinic here in the United States and some other countries around the world, which were interested in helping make sense of the actual epidemiological models. And so we dove into that a little bit as well.  And early with the pandemic, we had a model that performed better than the UPenn and the Oxford UK models for a while. And again, it wasn't just for... There's two reasons you construct these epidemiological models. One of them is stare at them and say,  "Look how bad it could be." Right? That's interesting, but not useful. The more useful thing to do is to say, "Well, what does this mean for decision-making about whether or not I need to open up a thousand person hospital." And that's the things we were with XXX Clinic and the state officials in Ohio, they had real decisions to make, how is this looking like it's going to move around in our community based on what we know and what would be the triggers that would start to tell us.  So we did a range of ensemble modeling methods for epidemiological models. And, you had a worst  case, a bunch in the middle and then best case, and what they were looking for were triggers. They  wanted to know, how can we tell as this thing moves along, which one of these model paths were on.  Because we've already gained out because we did the modeling, what our response needs to be in each  of these seven different possible ways this could go. And so for us, again, it was all about the decision. It  wasn't just making a model and drawing a curve. It was, how does that curve or an ensemble of curves  help somebody, whether it's XXX Clinic or a state government, how does that help them  understand what trajectory we're on? And then that starts to tell them, "Okay, in two weeks, given this  trajectory, I'm going to need this many ventilators and ICU beds in these places most likely."  So it was really a lot of the early work was around quickly understanding where the spread could go in  terms of the models and how could we tie that to rudimentary decision-making like, I have a bunch of  masks in this part of my state, and I may need to move them to this other part of the state, which is  certainly far short of optimization, but it's a poor man's optimization. So a lot of visualization of data, a  lot of early, what if, where could the epidemiological models go?  So I think the questions were... A lot of the media is interested in, well, here's the curve and here's how  bad it could get, and really whenever we do any work with data we always... I'm sure you think this way  as well, we advise our partners or customers to start with the end in mind, old-fashioned Stephen  Covey, 7 Habits of Highly Effective People, start with the end in mind and work backwards, right? What  decision can I not make right now? And I'm going to need to make it, or I need to make it better, or  faster, or cheaper, right? And then work backwards. And so the questions were, "I'm going to need to  know, do I have enough PPE for my frontline health workers? And if not, where can I source that? Do I  have it elsewhere in the state or in the country? Or can I get it from somewhere else? Or am I really in  some other jam where I've got to either source it externally or rational it." Right? So the key questions were around, how can this modeling help me forecast what my PPE requirements are going to be. Similarly, how does it help inform other medical resources like hospital beds, the Cleveland Clinic was trying to decide, do we open up a thousand bed hospital next door or not, based on where we see things going. The ability to look for triggers to see which of these range of outcomes are we progressing on at the moment? And that had two questions attached to it. What are the implications for my response capabilities?  Respondent:  And there are great models for those. And I have not seen anybody put together an honest model that  tries to balance those and find a minimum, right? So you have politics on both sides, right? You can't  shut things down. People got to work. Now on the other side, it's one, death is too many, right? And so I  think the real middle ground is somewhere there's a push and a pull. And there's some place in there  where you've got to have some balance, right?  So we have to plan for that, it's going to impact our ability to provide benefits and services to our  citizens. So we've been asked for all of that, and then we've been asked on the public health side, help  us track and understand the epidemiological spread, contact tracing, medical resource optimization. But we haven't been asked to put that together and create these value trade-off models. And I think  presidential election year, maybe we would have had more of that conversation in the United States. I  don't know, but that's the... The real answer to the American people is there is some level of public  health impact that is irreducible, right? You could trade off all of the COVID deaths, but you'd have  increasing challenges in other places, right? You can even run out the fact that people miss six months of cancer treatments, you can run that out over the... I'm sure you guys have done all this.  Respondent:  You could run that out over the long-term, and there's a fatality rate from that. So all of that should be  put together for an honest conversation with the public. And we haven't seen a lot of interest in  connecting that up. There's usually we're interested in opening up as quickly as we can, we're sensitive  to the public health impact, but we really need to get people back to work or it's, we've got to protect  the lives of our citizens, and we'll try to provide stimulus to get people through and economic support,  but we're really interested in driving down the public health impacts from COVID.  Respondent:  And I think the nuanced, the leader that I would love to have is the one who would be able to put  together that balanced picture and then tell that story in a way that was honest and...  Interviewer:  And I think it's been interesting, that COVID has brought to light big simulation models as an item in the popular conscious now. So I'm curious from your conversations, the ones you've had, how do you think the pandemic, COVID, will change modeling as a science, it's applications, the hesitancies around it and so forth? For decision-making.  Respondent:  Yeah, that's a good question. I mean, models are... I would say most decision makers, certainly in senior  positions are just not used to dealing with model outputs. And in particular, they're not used to  challenging them. They don't know how to scrutinize them for where the model is strong and where it's  not. So I think it's good that these models are, of course being talked about a lot more in decision-  making, but there's also a lot of dangers there because you could tune the assumptions on any of these  models to get sort of whatever answer you want. So I think decision-makers have probably a ways to go  before they really know what to do with those.  Respondent:  Again, I know I keep coming back to XXX Clinic, but it really is, I think, our best story, because  those decision makers knew, okay, none of these models are right, but we can create upper and lower  bounds, right? That we're pretty comfortable at. It's no worse than this because the assumptions would  have to be so crazy that it's no worse than this, and it's going to be no better than this. So then the  question becomes, "Well, how do we know where we are in that continuum as this thing progresses?"  And we sort of game planned for a number of potential outcomes. So models are better not for  predicting the future, but for what if analysis for decision makers.    So these models would say, "We're going to have 2 million deaths in the US by," whatever that's sort of  not a helpful... And it also can make models look like they're untrustworthy, right? Because when that  doesn't happen, you can say, "Well, see models are garbage," but the better use of those things for  decision makers is to say, "Well, how can the models show me a range of possible outcomes, given a set  of inputs? And then how can I understand how I would plan for those outcomes?" Right?  For those models. What decisions would I need to make if any of these five scenarios came true? And  then as things are progressing, I can watch and see which of them looks like they're coming to pass so  that I can start to activate those plans. That's the much better use of modeling. I think that's probably a  little bit more nuanced than most people are thinking of them at the moment.  Respondent:  There's been modeling for years about how to do this in influenza pandemics, right? So, ring  vaccinations versus other things, et cetera, et cetera, et cetera. So what the models can help you  identify geographically, and by cohorts in a population, who you would vaccinate in what order given  the quantities you have to minimize certain impacts, right? Public health impacts, right? So you might  say, "Well, we're going to vaccinate frontline healthcare workers and highly vulnerable populations,  immunocompromised, and nursing homes." Right? Then from there, what? Right? How do you roll it out from there? |
| H3 | Respondent:  Oh, yeah. Well for one, we were typically seeing ourselves about two weeks behind New York City, the   West coast, Seattle, the major cities that were really blowing up. So anything that we were seeing was   we were following what the data was showing from other centers that were more impacted. So that was   what's being used to model. So we were taking those things very seriously, not waiting to see if it would   happen. It was more of a question of when was it going to happen to us, when were we going to see our   spikes? So what were we going to do ahead of that? So that really kind of drove with why we made the decisions we did. So we were looking at more things to avoid and prevent, and what were going to be the factors that were going to limit that? So the stay-at-home, we knew that if we could do that, that was going to perhaps drive down speed. Promote masking, PPE, social distancing, all those things were driven based on the data being seen in advance of what was happening.   Interviewer:  So from what you had discussed, it seemed like there was a lot of looking at other states   and what was happening to them. How much of the data were you looking was internal to North   Carolina or even internal to the healthcare system that you had privy to?  Respondent:  Yeah. We did work with individuals at UNIVERSITY who did the projections, some of the epidemiology or a   specialist who had that, and we coupled that with the notable models that were used on the national   scale. So I think there was one out of 10 state, and some other locations. So we probably had about five   different models that covered not only in our state, and our local area and also compared that to the   national level. So we aggregated it all. We weren't going to just isolate or pick and choose certain things.  I guess as far as my understanding is of what we knew, we knew that we just needed to do that. So our   department, our team was very data-driven. We used data to define a lot of stuff, especially for doing   six sigma work, and those types of things. We do have people who that's in their space, that's their   wheelhouse. Because we didn't know what to expect, it was intuitive that we had to go to data. And if   models and everything were projecting certain things, we latched onto it. So it was clear on the news,   we got people who do those curves. Some of it is past experience. From previous N1H1, those types of   stuff, Ebola, looking at what would happen if Ebola came. So we were Ebola-ready if it should show up in   our areas. That happened a few years back. So we kind of had those mechanisms in place. It was just matter of, "Oh, Penn State has a national model we could use? Let's grab it. There's a national model here that's top of the line, too, let's grab  that."  Interviewer:  Do you think among those models, or just other data that you were monitoring, there were any key data   points that were more closely monitored in others. And I think that could be considered from the   epidemiology of the disease in terms of how many cases are bumping up, or deaths, but also hospital   capacity and supply and stuff like that. What were some of the indicators?  Respondent:  Yeah, yeah. So obviously, the epidemia, would show the rate of positive cases, and  then obviously, if we looked at our region... So if we get our region specific, that helped us indicate us  whether if they said by July, we're going to have XXX hospitalizations for COVID, what does that look like to our capacities for the health systems. So amongst our nine hospitals, what do we have based on the number of beds, based on the acuity to address certain acuity levels of folks that have COVID. So some people are minor. Some people are really sick. So now that leads into various levels of operations. Do we have enough ventilators? Do we have enough  beds? Do we have enough respirators? All these types of things goes down. So not only to equipment to  care for the high acuity patients, to the PPE associated to care for them, what is our PPE stock level look  like to manage those ICU-level, and it just cascades down. So we've got operation models associated to the epidemiology, and then operation models help to indicate the supply chain logistics side to support that. So with that, we are feeding the supply chain inventory data to forecasting. So there was a model in forecasting supplies and inventory, and then all the operational reports; bed management, in patient cases, team members who have got positive for COVID, what our staff, all that kind of stuff. And then obviously we got kind of a two-way flow from our team member base. So we're pushing information to them, but we're also getting information back.   Interviewer: If you had a capacity to develop a model to help answer a question that you guys are considering, what   would it look like, or what would it answer?  Respondent:  Good question. Very good question. I can't think of anything on the head. The models are the models.   They kind of give insight. We're always looking for better models that might be more accurate to   understand changes, but the real deal is is that when we look out in the medical side, my wife's a   physician. So she is a critical care physician. So she works in the ICU. She works in the COVID unit, and   she's an emergency medicine physician. So she sees it as they come in, too.   Interviewer:  You had mentioned earlier in the first set of questions that you had been looking at different states,   models, and even some data that was coming in from North Carolina. I wonder how much is that kind of   science, if you will, being programmed into decisions right now. Are you still monitoring these models?   Do you have your own models set up to help evaluate decisions?  Respondent:  Yep. Yep, those things are still ongoing. The models still indicate some things, changes in season,   changes in things. When the school year opened, does that affect things in what we can see and   represent. So absolutely. I don't think any of this has gone away. We still have a PPE taskforce. We may   not meet as often. So as things arise, then we will reconvene to do certain things. So we've established a   quite good foundation for operating under the pandemic. Some things are going to stay as-is. So   screening doesn't go away, the models don't go away, to make operational decisions, all of that. |
| H2 | Interviewer:  Maybe just as the last set of questions and the last three or four minutes we have. When you think   about these different decisions, and you can either pick one or talk about them generally, what kind of   authoritative sources are you guys deferring to or pieces of information that have been helpful or not? I   guess, the spirit of my question is that COVID is a dynamic changing, confusing context. And so we're   just curious how you guys have tried to make sense of it and the authorities or information or evidence   that you…?  Respondent:  Yeah, so that's a great question. So what we did is we developed using a, I guess, a tracking tool from   the University of XXX. They've got the XXX center there. So what we did is we took some of the   work. And my brother is the executive director for all of that. So anyway, we stole their work. So what   we did is we created a COVID 19 tracker that looks at the State and then it divides the State and the   regions, and it looks at what's going on. And essentially it creates a dashboard where we're able to look   at what's the disease velocity, so how quickly is it spreading? And then it developed some trend lines so   we can then develop some assumptions that drive our actions in regards to how we're supporting our   members. So, our dashboard looks at the velocity of the disease growing new cases, deaths. It creates the disease   index trend. We look at resource utilization and how quickly that's being consumed or used. And then   we split the State into, essentially, seven regions and we track what's going on regionally because that's   how the virus acts and that's how patients move around. And people move around the State. They   usually move around by organic geographic regions. And what we found is the virus essentially cycles   through these different regions. |
| PH1 | Interview:  I'm curious, maybe this is a broader question just right now, but to what extent,  any kind of like modeling work has been involved in these decisions? It's been funny, I have been in the world of simulation models for a little bit, and it's really just been my world for a while, and suddenly everyone knows what a simulation model is, and I can see the graphs and the nice curves and so forth. I'm curious how those tools have been involved prior to the decision-making processes or even right now going forward.  Respondent:  I would say as far as vaccines and everything, I've not seen one, but in terms of simulation models when   it comes to COVID, and opening, expanding, and contracting, I appreciated that because early on, being   a big county, we also have like a big county high-impact call every Monday. State epidemiologist, Zach   Moore, shared that modeling with us, and pretty much said, "Hey, here's what happens when you enact   stay-at-home orders." You could see it going down. "Here's what happens when you open it up." All of a sudden we see it go up. "Here's what you need to  do if it gets too out of control." Here you go right here. I mean I still have that in my office [inaudible 00:43:42], but I appreciated it because even though it was the first time I seen it, it made sense. I was like, "Okay, we can see." For me, the goal for me was like, "Okay, how do I get it to where I don't have to expand and contract? I can slowly open it up." You get to the point where the curve just kind of like flattens out, and it even eventually starts going  down. Of course, the overall total cases is going to continue to go up until a vaccine is, but that seven-day moving average is what we had to really teach basic core modeling, we had to really teach our residents like, "Don't pay attention to that because that's going to keep going up. Here's this one that  you really need to know because that let's you know how the prevalence of COVID is really tempering up and tempering down, and you want to see that seven-day average go down. It lets you know that community spread is low. It lets you know less people getting infected." The modeling played a key role. Again, like I said, with the different tools that we have from being able to graph who's getting infected to being able to show them by zip code what their infection rates look like. I mean it's paid dividends. |
| PH4 | Respondent:  So, I will tell you, we didn't have a lot. We were looking to some models from, oh. I can't believe I'm   drawing a blank. Was it the Washington State or-So that's the model that we were all looking at early on, but then I would say as North Carolina put some of their mitigation strategies in place, you may remember Secretary Cohen talking about "flatten the curve," and then partners over at UNC, they had their projections around when we would see our  surge and peak, which they said would be September, but hit us a couple months earlier. So, I think none of us had solid models to follow. Again, it was like we were just taking this day by day and looking   at, "Well what's going on in our community?" You just remain flexible and you say, "Well if this, we got   to pivot." I can remember trying to staff up and hire enough investigators and contact tracers. You never   knew because one minute you're prepared and you want to bring in this any new folks, but then you hit   this lull and you're like, "Oh, well our case count is kind of down the last week or so." You plan and you remain flexible for the surge when it comes because you never know when it's going to hit. So, yeah. We didn't have any, in my opinion. I mean they were good, they were helpful. They gave us something to think about in our planning, but I don't know that anything was accurate.   Interviewer:  I mean looking forward, and I ask this very transparently as we're working with people from NC State who are trying to think through different policy questions through these models. I'm curious if you had the capacity to work with some of these folks or ask them, "Run this simulation, run that simulation" for either the state or perhaps more specifically XXX County, what would be those requests, and what kind of questions would you like to answer with that?  Respondent:  That's a good one. It's like a Christmas wish list. I would love to see the simulations, and this is what I think everybody would love to see, what happens  if we get to a space where we fully reopen and we take the limits off of some of the occupancy/capacity.  What happens if we open that back up? Would it be enough to do the masking and the hand-washing?  Probably not. I'm curious what it would look like. Well kind of, I'm curious. I think I know. I was going to  say, with school, if we were to reopen in the spring and all 19,000 students or so were to come back again and be on campus, what would that look like? But, I think for a lot of these, I feel like I know, I   think I know, what the answer would be, so maybe with the simulation and modeling, it would take into   account if you had immunity amongst this percentage of the population because to me, maybe that   would give us some goals in our vaccination planning and programs. "Well, if we get to this, we stand a   better chance of this outcome." So maybe that would be my request if they were doing some kind of simulation and modeling, would to be figure out the percentages of immunity if we were to get it. How would that change the way this  virus plays out?   Interviewer:  Yeah. Any other questions you'd like to answer with it?  Respondent:  Gosh, I'm sure my business partners from the chamber, they would love to see what would be the   impact of revenue if we could open up some of the other sectors that are still currently closed as a result   of having some more immunity. Yeah, it's a ton. Oh gosh. This is endless, right? |
| PH5 | Interviewer:  Absolutely. So as I mentioned earlier, we are working with some folks from NC State in particular to help   try and model different aspects of the pandemic and so forth, especially North Carolina. So I'm curious,   if any of those simulation models have kind of become more popular during this pandemic were at all   used during the early decision making process, and if so, how?  Respondent:  Those were incredibly controversial. Well, the projections ...Number one, like I said, we've got a very active media here. And a lot of them like to think of themselves  as armchair epidemiologists. So they would take our numbers and they'd try to do interesting things  with them. And why are you looking at this model and not this model? And what is it about this model?  And so there was a lot of that going on. People really ... And we're seeing the same thing with metrics right now. People want to see something that they can hang on. They can say, okay, this is what the model says. This is what's going to be accurate. And some of the models were so far apart that it was really challenging to come up with something specifically for Mecklenburg County. And that's ... The other model, the other problem was a lot of the models are at the very best are state level models. They're not local. And because we looked pretty different than the rest of the state in terms of numbers and those sorts, they wanted to see something from here. So it made it challenging with the models. Both of our hospital systems were doing some of their internal modeling, but they also weren't real comfortable sharing a lot of that. We finally have gotten to the place where they will share their modeling, but it's not something that we put out. It's more helpful to us in terms of thinking through things. And we did some work here looking  at the different models, and put that out ... Oops, sorry. Put that out a couple of times. But fairly quickly got away from that because it was not being helpful in  terms of the conversation we were having in the community and what needed to happen. We focus more on the numbers that we were seeing here, the metrics we were seeing. And the impact that we were seeing from people wearing masks or not wearing masks and things like that.  Interviewer:  Right. Well, so maybe to pivot a little bit to the scientific then, and I ... Again, we are working with these   NC State engineer modeler people. And so I'm curious, as I speak with them about the conversations I'm   having with folks like yourself, what decisions are being considered, if you had access to one of these   models and had the kind of technical ability to adjust it and help inform any kind of policy that's on the   table right now, what would that be and what would that look like for you?  Respondent:  That's hard to say right now because I've got to tell you, one of the biggest challenges that we're starting   to run up against is access to good data. Right now if I look at the cases that come through that we have   information on, 37% of them I don't have demographics because even though the state has required the   labs to report demographics, it's not happening. And so I can like I said, 25% of the cases are among   Hispanics, but 37% of all of our cases I don't have the demographics on. It could be a whole lot higher   than that. It could be lower than that. As we move into point of care testing, especially with the antigen   testing, there is no lab report that comes out of that testing.  Respondent:  So it's not reported to the state. We are working with those that provide that testing in our community.   And as much as we can we're getting the test results, but we're not guaranteed that we're getting them   all number one. And we're definitely not getting the negative test results. So it's hard for me to think   about modeling built on bad data. We're having conversations about whether the metrics we've been   using are metrics we need to continue to use because I'm not sure the data ... Whether I have   confidence in the metrics that we're going to push out. It's really hard because our community is ... If we   try to change the data we're providing, or slow down on putting it out, man, it becomes a huge issue.  Respondent:  And if it wasn't a huge issue for the community, the media makes it an issue for the community. So   that's the biggest challenge that I have right now in terms of thinking about modeling.  Interviewer:  Absolutely.  Respondent: If we had good data, modeling would be great. It would help us look over time at what's happened here   in LOCATION to predict what could potentially happen moving forward based on changes that we're   seeing and what's reopening, how many ... What percentage of people are actually wearing masks.   There are all sorts of things that could feed into that modeling if we had good data. But it's a little   challenging right now.  Absolutely. Yeah, we're trying to ... So, we're trying to do this model for ... It's mostly the whole state.   And so the extent we can get [inaudible 00:40:44] level data has been super helpful, but it's not super   easy.  Respondent:  Yeah. Well, and I will tell you that it's hard for us. We look at the state data, but we look more at our   local data just because we don't look like the rest of the state. So that's ... The modeling for me would be very helpful if there was a focus on what's going on here. It's important for me to know what's going on in the state. Don't get me wrong there, but at the same time, it doesn't help me to look at the state and see that their numbers are going one way and mine are going the other.  Interviewer: And so I'm curious, as I speak with them about the conversations I'm having with folks like yourself, what decisions are being considered, if you had access to one of these models and had the kind of technical ability to adjust it and help inform any kind of policy that's on the table right now, what would that be and what would that look like for you?  Respondent:  That's hard to say right now because I've got to tell you, one of the biggest challenges that we're starting   to run up against is access to good data. Right now if I look at the cases that come through that we have   information on, XX% of them I don't have demographics because even though the state has required the   labs to report demographics, it's not happening. And so I can like I said, XX% of the cases are among   Hispanics, but XX% of all of our cases I don't have the demographics on. It could be a whole lot higher   than that. It could be lower than that. As we move into point of care testing, especially with the antigen   testing, there is no lab report that comes out of that testing.  Respondent:  So it's not reported to the state. We are working with those that provide that testing in our community.   And as much as we can we're getting the test results, but we're not guaranteed that we're getting them   all number one. And we're definitely not getting the negative test results. So it's hard for me to think   about modeling built on bad data  Yeah. I think as I mentioned earlier, one of the biggest challenges has been lack of consistency. Whether   that's guidance, whether that's data use, whether that's modeling, whatever it happens to be. And as   we go into discussions around vaccine, that consistent messaging is going to be huge or disastrous   depending on which way it goes. So I think modeling is ... The work that they're doing is critical I think as   we move forward on this because it's not going away anytime soon. And it would be incredibly helpful,   but it would need to be consistent. And we need something that's clear, easily understandable by the   general public. If it's going to be shared with the general public, and believe me, around here if the   media gets ahold of it, it'll be shared with the general public.  Respondent: So clear messaging, consistent messaging, consistent modeling and good use of data. Those are sort of  the main things that I think are critical for us. |
| PH3 | Interviewer:  Yeah. I'm curious. So like I said at the beginning, part of our group is doing a lot of these simulation   models, which you may have become more familiar with during COVID. I'm curious, what influence if   any of those kinds of models had and the decision making process early on for the health department?  Respondent:  Well, early on I mean, our first case was in DATE. Obviously, we started hearing about this in late   December, January in China. But then we were looking at the Johns Hopkins map, and we could see the   cases on the West Coast, in Washington State. And we definitely were seeing that and I remember   telling our board, our Health and Human Services Board. "It's not if, it's when. It's not if we get any cases   it's when." Because we're the fourth largest county and the eighth or ninth largest state in the nation. So   we're going to have cases. This is not some county in rural Wyoming. I mean, we are in the LOCATION area right beside XXX County, [blocked for confidentiality], so it's going to happen. But yeah, so   definitely, I think those models play into it also. I mean, we're looking at it now the state metrics on their   COVID dashboard. We're seeing for a while, we had more cases in LOCATION. But now, LOCATION’s cases   and deaths have increased above ours. And we're seeing more cases in our neighboring counties, while   things have slowed down here, we're seeing more in others.  Interviewer: Looking forward and trying to  make decisions, whether it be for vaccinations or the school reopening. Have any of these kind of projection models been helpful, to help you get a sense for where things are going to be in three months or something? Or how they've been used in a conversation?  Respondent:  Well, originally, we focus on a model out of the University of Washington. I think it was the University of   Washington, isn't it? That was one of the hospital CEOs we're really looking at, and they lobbied Governor Cooper, for the stay at home order. And so that was very effective. But I think we were all thrown off guard. We thought   in the summer this will slow down, which is not the case. |
| PS7 | Interviewer:  Yeah. No, that's fair. I mentioned at the beginning that part of our group is these folks at NC State who   are industrial engineers but have been doing various COVID modeling work. I'm curious in the   conversations about whether is bringing back students or not from spring break or otherwise like, how   those models were used in the decision-making process, if at all?  Respondent:  Yeah, they were. I'm going to divide it into two categories. The way NC State approached pandemic   response, let's go through second quarter and into third, and then morphing a little bit. We used models   that were both local, national and international, and it was kind of two camps. So our medical director is   also a faculty member in hard sciences. I don't diminish anybody's stance or where they work, but hard   scientists, they like to talk hard sciences.  Respondent:  So we had several faculty think tank groups that came together to look at data modeling, but it never   extended really in the conversations to what we could do as a system because of a lot of higher level   expectations from external participants to the universities. So like board of governors decision. And   good intentions using frameworks and models, both the local impact, the North Carolina impact, the   national to the international. So scaling it on four levels. The university, when we made decisions, we   really base things off of twofold. If it could be local impact, meaning county and state, that was our first   focus because that's really our stakeholders' target audience. The 20% of other out of state or   international, there was this bubble that they weren't diminished, but they weren't priority one because   at that point things were a little bit out of our control. So the models that were put together (silence)   but they're all in the same space.  Interviewer:  Sorry, I jumped out. Sorry, you had said the models that were put together and then it cut, and then I   dropped.  Respondent:  It's okay. It's all right. So the models that were put together were really two camps and one was more   faculty centric, and one was more, I'm going to just call it staff because that's probably a really good way   to delineate that. The faculty models used all the hard science, the community spread data, the systems   data really looking at what's the probability of impact here. So viral loop, positive rates, mortality rates,   things that really impacted some decisions. It met in the middle to the staff side or the operation side,   because as we talked about human behaviors, then that informed gatherings or de-densification, or   talking about modeling our health and safety compartment, talking about how the best to do that.  Respondent:  Before these were the new fashion thing. That was how many people you put in a room and the systems   from that perspective, how does HPAC suck it in and blow back out? I'm going to lightly use the word   more esoteric conversation on the models and faculty and research side was translated to a very   practical, put some barriers here. Don't do anything here. Open the windows here. Those two groups   intersected quite substantially. University as a whole didn't necessarily see those as official groups. They   are our day jobs, let's put it that way.  UNC counterparts, we talk hand in hand with counterparts from all the other schools and UNC and ECU   being comparable size. There were programs that we brought in here as a non-medical school facility   that our two counterparts didn't move as fast on. So I think some of it is the right people doesn't mean   that their day job ever thought they would be pandemic people, but for the right thinkers. So the   modeling is important because if you have the models and the frameworks to build off of the right   people who can triangulate, in this case, can make them happen. I think we were a little ahead of the  curve, maybe step every day, because we were having those types of conversations in order to make it   easier.   So XXX is my parallel peer, pretty much like COVID Queens. She owns anything medical, public   health, and I own anything operations and logistics. So our jobs have to compliment one another, a   hundred percent. So if there's an idea for a surveillance program and she's 2% of a population, then I   have to help figure out how to get the tents and the traffic and the rest of it going into her surveillance   program. And so we spend a lot of time and have for six months now creating intentional intersections.   You know there's going to be a need to make the think tank happen in reality. And that's how it's the   best intersected. It's entertaining though, because the two of us were both faculty. So where she's   faculty in a hard science, I'm faculty in the college of education. And the way we approach and the way   our conversations go was predicated on our role in faculty.  Respondent:  So we were privy to hearing more of that academic continuity and academic decisions for faculty, where   if you had just the two of us in staff roles, we would have missed a third of the triangle. And so it really   became that we were very fortunate to have the capacities that we do in our day job, plus second job   plus faculty. That we could create a very strong triangulation of faculty and employee in an operations   sense. And I think because of that and the intentional like, extremely pick up a phone, I'm thinking about   this, we're doing this, where she ran more of a research modeling group to look at the models from a   public health perspective. And I could do a similar correlation on models where it came to operations   perspective. I say this without calling it bragging, but I think UNIVERSITY was ahead of the curve on several   things, because we were able to do that.   Now's our time to look at the epi curves, to go back and look at the models, to go back and look at what North Carolina as a whole or domestically we're talking about and making some decisions. We're going to balance the two again. We're going to make decisions and policies can tell us how to make decisions. There's good intention in a lot of decision-making, but it can never come to fruition, a myriad of reasons, and we just have to be realists. My word is authentic. You do as best you can, based on the circumstances.   Again, we work with these modelers people. We're trying to think through what other questions we can help ask with it. It's been developed over, honestly, the last decade using different infectious disease models. And so we're   thinking of big questions that we can be useful for help answering. And so I'm curious if you had the   opportunity to play around with one of these things and create an environment in which you can test   certain policies or something like that, what would you use it for? I don't know if you've ever thought   about this.  Respondent:  I'm spending an inordinate amount of time trying to understand how people make their own tweaks. In   public health perspective, my dad calls me once a week and he says, "What's the latest?" You know   what? You can read. Here's the MMWR. Here's five or six things. My litmus test for any type of person   who's in the public health world and looking at epidemiology and everything that is to be absorbed from   the situation, it would be helpful to understand the human behavior, reality of truth, how its impacted   the success of sewing the curve or flattening. Because I think that a lot of the conversations that we   have now are complacency driven. Like people are over it. They're done. It's no longer impacting them   personally. They don't know somebody who's sick or sick enough to be hospitalized or dying.  Respondent:  And so they've moved on, this pandemic is over. When you look at Europe, then it's resurging. Or you   look at how flu season is going to come in and people go, "Oh, don't get a flu shot. I don't believe in flu   shots." That would be the part that I would think the most helpful is to more understand the human   behavior side because the system, when we do systems modeling and we look at data, it's super good   until it impacts you personally. And then you're like, it's not me. It's not me. |
| E7 | Interviewer:  Yeah. Were there any... so maybe one of the last questions in the science space. Like I had mentioned,   we're working with some folks from NC state to do a lot of different, like infectious disease modeling   and projecting, again, where the pandemic could be under different assumptions of mask usage and   vaccination and et cetera. I'm curious if any of that kind of modeling work was involved in the decision-  making process? Whether back early on in the pandemic or more recently?  Respondent:  Not a whole lot. I mean, we talked about using or looking at positivity rate, and then trying to, but we   worried, our concern was, if you say, well, we're going to choose 5% plus or minus 0.1 to 0.2, over above   a certain number. What if you say is 5% and you're at 4.9? Do you close or not? Or you're at 5.1, do you   close or, if it was 5.1 over here in apex and we're talking about closing schools and [inaudible 00:26:36]   Or sort of roles via, which might be 30, 35 minutes away, on the other side of the County. So, we kind of   stayed away from trying to put ourselves in the corner, so to speak, that honing in on a specific data   point and have you, but in a district of our size kind of, taking in more information than just more data   than just hanging in decision on one data point. |
| H5 | Interviewer:  And so, I'm curious during those first few weeks, few months to the extent those   kinds of modeling conversations were involved in the decision-making process, and if so, how?  Respondent:  Yeah. So, we quickly got a little group together. I called it the think tank, where it was just a group of us.   I don't even remember how we got started really. NAME, and NAME, just a bunch of people. And boy,   I'm blanking on everybody's name right now.  Interviewer:  That's okay.  Respondent:  The other NAME. And we quickly got together and just started spitballing ideas and throwing ideas around   about what could this be? What does this look like? How would you communicate this? And that was   really valuable for me. Again, because I had come from public health where it's a team sport, right? You   don't do anything alone. And not having that group of five that you hung out with and bounced ideas off   of and just chatted through something was really, for me personally, one of the most difficult parts of all   this.  Because I felt the weight of the responsibility myself. And I didn't have my buddies in public health to   say, "Hey, Aaron, what do you think about this?" So, having that group was really valuable. And then,   trying to look at the models and models are different and yet they're all the same, right? So, looking at   the Washington University model, looking eventually at the Hopkins data, those proved to be very, very   valuable inputs.   Interviewer:  I'm curious, sorry if this is too abstract, but what really was the incremental value from looking at some   of those models? Otherwise, to say what do you think you could not have said about the pandemic or   about how to prepare for an UNIVERSITY that you could have said with these models?  Respondent:  It was the visualization. It was showing a graph of how bad it could be. That was the…You just owned the aesthetic value. Yeah. Well, we're all adult learners. We all learn from it's all visual cues. And it's not abstract. I mean, it is abstract, but it's not abstract words, it's that hard stuff you can hang your hat on. I mean, I think that   that was probably the most valuable thing. Everybody used to laugh at me and said that I had graph   envy over the state health department because she would get up and show better graphs than me. Of  course, I'd be [crosstalk 00:26:05]. But anyway, it's that visualization and just putting it in front of   people's faces. Because otherwise, it's all words. And it's clients based. Because we're, after all, a TYPE of school. So, you need that data. You need that science base. You need a graph, you need a chart, you need a pie chart.  Interviewer:  Yeah, absolutely. And so, again, similar question as I asked earlier, the use of these models and making   decisions, and I'm curious, one level, to what extent any model is currently helping you make these   decisions, or to the extent you want to entertain this. If you had the capacity to use a model to help   answer a question that you would like to have answered, how would you use it? What question would   you ask?  Respondent:  Yeah. I'm going to reveal my bias about models, which is honestly, it's not high. I've worked with models   for a long time. And models are only as good as the comfort level you have on the data going into it and   the assumptions being made. And unfortunately, I know enough about models to make it dangerous for   me and that knowing that if I change one little variable a little bit, I can predict the end of the world or   the beginning of life.  Respondent:  So, yeah, I'm always skeptical of models. I don't know that models are going to tell me... they might tell   me the what if situations, but unfortunately, what we saw was that the models didn't predict what   happened to us. They can't predict people's behavior.  Interviewer:  Is there any information that, again, you're not a modeler, it's not going to put you in a spot necessarily,   that you think it'd be more helpful into consideration to make them more accurate, or the extent you   can speak to that?  Respondent:  I've worked with models for 20 years. And I've never had one that I would bet my practice on. I mean,   Alun Lloyd and I go around and around on this all the time. I think they're helpful, for me, they predict  worst case scenario, which is, of course, sometimes what you have to plan for in a disaster pandemic.   So, they help you predict what the worst thing that could happen given the assumptions that you have.   But it could always get worse. It didn't predict what happened to us.  Interviewer:  Yeah. I want to nag you on your philosophical commitments to modeling or not right now, the last few   minutes we have. No, no, no, that's interesting, personally interesting.  Respondent:  I mean, they fascinate me. They do fascinating. I love listening and go, "Wow, this could happen, that   could happen." But at the end of the day, they're not going to tell me what to do. Because here's the   analogy. And again, I learned this a long time ago in medicine, statistics apply to the population, they   don't apply to the individual. And the same is true if you come to me and you say, "Doc, how long do I   have to live?" I can tell you, "Well, 90% of the people have your illness die within six months."  Respondent:  But I can't say that you're going to die within six months. And the same is true for modeling. It can tell   me that given these parameters, this is what you're going to have, but it doesn't tell me that I have   those parameters. And it doesn't tell me that those parameters aren't going to change. |
| E6 | Interviewer:  Yeah. So this is just the last few minutes. Some of the folks we're working with are from NC States, they  do a lot of infectious disease modeling and projecting kind of different trajectories for the pandemic  under different kind of policy scenarios and so forth. I'm wondering whether any of that kind of work  has been involved in the decision making processes for the school system. So looking at different models  of how the pandemic has developed, how it's going to develop, whether just under different policies or  whatnot, or how has that helped if at all?  Respondent:  Maybe nothing with NC State. I know I've been working with, there's a group out of Duke, that's running  the... They've created something called the ABC COVID collaboration. They've got a couple of professors  there. I've got, I think LOCATION and LOCATION and LOCATION. And now several other school districts around  the state have kind of, I don't know, maybe hanging on to see what it looks like, see if we're going to get  involved or not, but they're sharing all their information. But I don't know if that's really the same thing  what you're talking about. I'm not sure if the information you're sharing is shared with health directors,  then the Health Director will share with us if it's something to look at, to be honest.  Interviewer:  Yeah. Maybe just to rephrase my question, not necessarily any particular work that NC State is doing,   but just the general kind of tools of models, whether it's created from North Carolina or otherwise to  help understand what the pandemic is going to look like, therefore, how to make decision in the light of   that.  Respondent:  I've not seen anything like that, except I guess maybe... [inaudible 00:31:38] if it comes through the local   health directors, he would share information with us. He shares studies with us. He's saying, "Here's   something you need to look at." Now that we're in Plan A, if there was some study that talked about   kids being a meter apart only decreases the potential spread by a couple of percentage points or   something. So we're focusing on at least being 40 inches away or so inside the classrooms as best we   can. And that's not always practical, but we're pushing the wearing the masks all day long, except when   you're eating. Really being tight with that. If there are some new models coming out... Sometimes, I'm   even afraid to watch the news because I'm not sure what it's going to look like….We keep track of it pretty close. My COVID people watch all that and the Health Director does send us  updates. |
| E1 | Respondent:  Well, we have admissions to the university, we have scholarships and financial aid, we do the record   system, a lot of other student and enrollment-related activities. Because of where we sit, we're big in   the data, big into matching the data around, and finding answers in it, so we're a go-to organization   from that perspective. We stay pretty plugged in on what's happening to other institutions, and peer   comparisons, and stuff like that as well. We got robust data, pretty good at modeling things, and things   of that nature, not necessarily COVID-related although we serve, plug in to COVID models we could find,   or somewhere like Julie Swann will share with us.  Interviewer:  What would've been helpful to have back then to help make some of these decisions, and whether that's data in the classic sense of the term or just other kinds of information?  Respondent:  I mean, there was a little bit, as you say, that we were already starting to look towards the fall, so we   had to start putting out things about ... Well, we had to do it for summer too, are we going to have any   face-to-face classes. Too dangerous. Why? Well, look at these curves. This is going to happen. Are we   going to change the fall calendar, and that has to happen. Well, now, it's happening later and later,   which is unusual but our normal thought is that should've been an upfront decision because it takes a   while for people to change decisions or plans. There was modeling, and we talked to people about ...   Chapel Hill and we did something similar. We started the semester early. It was an idea that once the   second wave come, and so there were models we would look at there, but it wasn't really hard data.   Interviewer:  We will get to that very shortly. I think just to round up the conversation of what was happening during   the beginning phase, I'm curious, you had mentioned very early on that your office is very data-driven,   and even using models to the extent they've been helpful. I'm just curious if you could speak to what   kind of data was used in those decisions, or what kind of models were most helpful?  Respondent:  Well, I don't think early on at that point there was much to model, truthfully, or we didn't have much to   go on at that point especially the decision for [inaudible 00:19:38] to de-densify the campus, send   people home, online, and not much a model can tell you there except that seems safer than ... Sorry.   That was off by a couple of minutes. Anyways, that you didn't have to model much. What we did have to   model was maybe what's going to happen financially with financial aid, or any of those things, and   repayments and those types of things. They weren't political in nature. They weren't polarizing   generally. They were debatable. Should you allow a student to change to pass rail grading after the   grade's been submitted? Well, people have different opinions but, at that point, people were pretty   cooperative. It's actually funny. As I just said that, it reminded me of this book I read about community   from a political scientist at Berkeley, and how communities can come together in a crisis and then, as   you get to different point of that crisis, how they can crumble and start having their own agendas. It   seemed familiar.  I mean, there was a little bit, as you say, that we were already starting to look towards the fall, so we   had to start putting out things about ... Well, we had to do it for summer too, are we going to have any   face-to-face classes. Too dangerous. Why? Well, look at these curves. This is going to happen. Are we   going to change the fall calendar, and that has to happen. Well, now, it's happening later and later,   which is unusual but our normal thought is that should've been an upfront decision because it takes a   while for people to change decisions or plans. There was modeling, and we talked to people about ...   Chapel Hill and we did something similar. We started the semester early. It was an idea that once the   second wave come, and so there were models we would look at there, but it wasn't really hard data.  One thing we did, for example, and our office is actually in a position where we have to take into   account a lot of political pressures. There's political pressures on how you admit students. There's   political pressures on how many students you admit, how you fund them, all sorts of things. We're fairly   sensitive to those kinds of things and try to mitigate risk and so forth, and we realized there's more   grade than there is anything else out there. There's not the perfect answer for a lot of these things.   Pretty quickly, we went to this company, I've been talking to them actually a few minutes ago, this group   Art & Science. They're a hierarchy consulting modeling type group. We worked with them early on, this   was back in the spring. We actually had our results about the first week in April or so, I think. We took   our admit and we created a pretty elaborate sample. They've got this pretty fancy technique of   simulated decision modeling or something. I forgot exactly what they call it.  Respondent:  We went out and we surveyed all of our admitted students and their families. We looked at in-state   students and out-of-state students, and stratified it to find out what are these kids thinking, and what   are they looking for from us? Are they worried about studying online? Are they worried about being   more than X-number of miles from home? Are they worried because their families have lost jobs? We   got a lot of that data and we actually bubbled that up through these systems to say we got to do these   things, we need this type of communication all throughout the summer. I think communication was a   big part of this. I don't suggest we did anything particularly well but ... Anyways, that was a big piece. An   example is, as a result, we created a massive online course that [inaudible 00:29:29], we got it through a   weird approval process, and it ended up being called Wicked Problems, Wolfpack Solutions, but it was   about COVID-19.   Yes, never comfortable being out there on your own, and so I think a little safety in numbers certainly plays into it. This is a collective decision-making process, so to speak, that the presence of higher education, the people governing higher education stuff, this is a collective decision, that this is probably the wisest thing to do. Quite frankly, universities are pretty good about generating data to support their arguments. We started seeing a lot of modeling. You're more familiar with them than me, but Julie had shown me a bunch. UCLA, at early, published some with hey, if this man's kid show up infected, how many kids are infected over the course of the fall term?  Interviewer:  Were you involved in any conversations in which those modeling results were a key consideration for   the decision itself, or is that something that you weren't a part of?  Respondent:  They weren't necessarily pivotal on this decision versus that one, but it's like ...We have a challenge of getting the faculty back in the classrooms because I think they're scared. On average, they tend to be older, and they have a lot of self-determination]. I think modeling would help about it. I think we need to be a lot more transparent with the data to  comfort the people. The question I was driving some of the stuff this morning and I got to admit when this first happened, and it started ramping up, I was a little bit scared. Now, actually I'm fairly   comfortable as long as I'm wearing a mask and I stay away from people, and I wash my hands when I go   through weird doors.  Interviewer: How could something like modeling work be useful in the decisions you're considering right now?  Respondent:  I think it'd be really useful. I'm a little worried we're not relying on them enough at this point in terms of   designing our testing, procedures or plan, isolating plans, quarantining plans as we bring students back.   Honestly, they may be as important in the ... important is we ramp up, but they're really important that   when we hit a difficult area, that we don't make the wrong decision there. Can we work our way out of   this problem versus, hey, send the kids home. How many kids do we actually have infected? How's it   growing? Where is it? You know, I've been impressed sometimes obviously I have to read a lot about it,   we can't pick up any [crosstalk 00:51:14]. The testing of the sewage water ... It actually is working at some schools reasonably well. I think we need more modeling to think about our testing plans. It is a challenge. I mean, it's probably even worse for the K-12. We do need to make this thing work. We have a challenge of getting the faculty back in the classrooms because I think they're scared. On average, they tend to be older, and they have a lot of self-determination [crosstalk 00:52:00].  I think if we don't get back there, there is a big economic downside. The modeling, I think, can really   help us mitigate that downside. [Inaudible 00:52:56] an actual, a better solution for ... It's not we're all   better, don't worry about it anymore, we're at the rose garden. It is we can make this work and we'll   make it work well.    Interviewer: What lessons have you learned on COVID-19 about how decisions are made in complex environments?  Respondent:  Well, for me, the COVID piece and really as we come to where we are now, it's just finding out or trying   to put any signs we're using or modeling we're using in the proper context, knowing its limitations and   so forth because everyone's using it as like here's the answer. No, that's a possible answer certainly, but   it may not be the answer, and what are the other possibilities of how accurate are these models. What's   your point of failure? In what ranges of circumstances are they useful and outside of those ranges do   they lose that? I'm still a bit puzzled on the whole thing. |
| E5 | Interviewer:  And so, I'm curious if while you've been part of the board and kind   of making these decisions about opening and closing, whether any of that kind of science has been   involved in the conversation of looking at some of the modeling that's being done to project the course   of the pandemic under different scenarios, or whether that is just not part of the conversation at all.  Respondent:  Our science collaborative, our medical informatics specialists have said behavior deprives outcomes.   And even as the metrics came through they said, "The metrics are the result of community action." So   where, and I think, you know LOCATION is fairly progressive in that way, and we've been pretty   good on mask wearing, all that stuff. And they said to us when LOCATION opens, when LOCATION opens, when   these others big school districts open, it's going to change the numbers, so get ready for that.  Respondent:  If we change our behavior, it's going to change the numbers. They also, and I mentioned this earlier, the   science collaboratives have said, "There is going to be COVID in your school buildings, period." The   question becomes, what are you doing to, but there shouldn't be spread sites, and there's a lot more   research that's coming out. NPR has a great article on this. I don't know if you've seen it, but they're   saying the schools are not necessarily spreading sites. They can be, if our procedures aren't working,   they will be. But if our procedures are working, they shouldn't be. They should be one of those safer   places. So those models could be helpful. But how do you factor behavior in those models?  They also, and I mentioned this earlier, the science collaboratives have said, "There is going to be COVID in your school buildings, period." The question becomes, what are you doing to, but there shouldn't be spread sites, and there's a lot more research that's coming out. NPR has a great article on this. I don't know if you've seen it, but they're saying the schools are not necessarily spreading sites. They can be, if our procedures aren't working,   they will be. But if our procedures are working, they shouldn't be. They should be one of those safer   places. So those models could be helpful. But how do you factor behavior in those models?  Interviewer: Assuming you can, and I do think you can, I mean, I do work with some of these models as well, and you   can account for that kind of by different parameters in the model of that estimate how close people are   in contact with each other, how many people are wearing masks, stuff like that.  Respondent:  Compliance is the issue, I think.  Interviewer:  Right. Right.  Respondent:  Right?  Interviewer:  Absolutely. So I guess, it seems like those formal models haven't been used directly in any of the   decisions that the board has considered thus far. Is that correct?  Respondent:  Not technically. I mean, we have the health department data, but it hasn't. So, no. No. |
| E4 | Interviewer:  So the folks from UNIVERSITY we're working with are in the kind of modeling space, so trying to   project disease, incidence and prevalence depending on different policies and so forth. And so, I'm   curious, firstly, whether any of those kinds of models have been useful, or you've used them throughout   the decision making process, whether back in February, March, or even currently?  Respondent:   No, we have not, unfortunately not to my knowledge. I'd say we've used information from the local health department, and again, looking at infection rates and things of that nature. |
| R3 | Interviewer:  So in this discussion, how much was like... I feel like I have to question, as a public health graduate  student, any data or measures or even like simulation models used in thinking about either option, from   a kind of quasi-scientific perspective?  Respondent:  So not for the big governing board, but for the reopening group, yes. Again, we had this advantage, so   Dr. NAME from UNIVERSITY  would literally bring the models that [they were]  running sometimes and say I'm   still working on this, but this is what I see. Your peak is going to be in October unless we start wearing   masks. And I don't think a lot of churches had that kind of [crosstalk 00:39:58], so we absolutely did.   Having NAME, who for all of the UNIVERSITY campuses, he's really overall in charge of infectious  disease spread in the hospitals, and really, to some degree, has a say in the care of all the COVID   patients that are in HOSPITAL SYSTEM.  Respondent:  He's not on all the campuses at the same time, but we had all of his knowledge, too, of what he was   reading, and what he was seeing, plus what the other two people on Mandy Cohen's staff were seeing   on a state level. So we did a lot of research. It was informed by a lot of public health knowledge. I never   made my own model, I'm not trying to say that by any means, but we could see them, because those   folks let us have a peek into their world. |
| G3 | Respondent:  We were real sensitive to the needs of our  community should we have a large outbreak, which everyone anticipated would happen back in March.  Talking about models saying in our community we'd have 1,000 cases in March. We didn't know at that time how we were going to deal with that. That was a big concern for us. In March, when all the fluff that was in January that it wasn't coming and we weren't going to have it   and all the stuff that was being put out. When it was apparent that this was coming and it was going to   affect everybody and it was a big deal, then we had to make some decisions. At that time we had not   had any cases in LOCATION   Respondent:  However, what we found was we didn't have to just deal with our population. We became the place to   ride out COVID-19. All of a sudden we had summertime visitor populations showing up in LOCATION.   Our system could not possibly accommodate that. At that time, we had estimates and models from   Washington University and all the others that were estimating thousands of cases in LOCATION  given   that number of people and where they were coming from, New York and Pennsylvania. Places where   they were really having big outbreaks. We have, as I said, 15 or 20 beds in our hospital. I think we have   12 ambulances. Not only do we have nowhere to put patients if we had gotten those kind of outbreaks,   we didn't have the ability to move those patients somewhere else.  When we have a state of emergency in a hurricane and we can't bring people in because we don't have   resources and facilities to deal with them because the hurricane damaged them and tore them down.   We close down and we evacuate. We don't allow people to come into the county.  All decisions are risk/reward at some level. That's exactly how we went about it. We felt we had the   legal authority until the Emergency Management Act. We were okay with that. We had some   conversations with the school and government to make sure we weren't missing something we should   have done. We looked at some case law, there's not much case law to help us there. But we felt   comfortable that we could do that. Then the question became, again, the risk reward. If we do this,   what's the risk? The risk is it's going to negatively impact our business community. We're going to get   some pushback there. The risk is the folks that want to come here are going to be upset and they are   our livelihoods. We appreciate our visitors, I think that's why we're in existence because they come here   to spend time and enjoy the beaches and things. We knew we were going to upset those folks as well   but when you looked at the risk side of it, if you believed any of those models that were out at the time,  the impact to the community, the health risk not only for the people that live here but the people that   we allow to come here.  Respondent:  When people are allowed to come here, we're telling them, "We have the resources to take care of you.   We have the power. We have the water. We have what we need to take care of you." In normal times,   we do. Even our 25 bed hospital is fine in normal times for the 300,000 tourists that come down here   every year, every week in the summertime. We know how to deal with that. Our system is built to do   that. But it's not built to deal with the numbers that you would have gotten if you believed those   models. The risks that we found were much greater than the pushback and the angst we were going to   create with some of the populations that weren't going to be favorable, if you will, to our decisions. We   understood that but felt like we could not take that risk. We didn't want to be standing there in June   saying, "We could have done this and we could have prevented those thousands of people stacked in a   room because we didn't have anywhere to put them." That pretty much made the decision for us.   Interviewer:  were there any other quasi-scientific authorities or scientific pieces of evidence that you're   using to make some of these decisions? Whether it would be individual, authoritative voices or data that   was being collected that was helping you understand the risk/reward trade off?  Respondent:  We have our health director, she's basically responsible and she's the information liaison if you will for   COVID-19. We, me and the board, we weren't out trying to vet the data or peer review it or any of those   kind of things. But our health director was taking the data she received from the CDC, she was taking the   information she received from the North Carolina Department of Health and Human Services, she was   taking the models that they were using to create the guides that they were giving. We took them to be   trusted sources. We read the media accounts and we read all that stuff |
| G2 | Interviewer:  Do you remember, all in the similar vein, whether there was any discussions... And again, this is either   for county decisions or decisions within your own organization, but you saw some of the projection   models and stuff that were being popularized back then, to the models that were projecting the   trajectory of the virus under different scenario and contexts, were those at all discussed back then?  Respondent:  Yeah, the rates, how the rates... We were sort of doubling, so the data on the case numbers and   hospitalizations. We frequently had folks from the hospitals that are in surrounding counties, on those   calls, talking about their availabilities and things like that, just the rate of positive exposures and positive   cases in surrounding counties. |
