# Supplementary material for: Use of Modeling to Inform Decision Making in North Carolina during the COVID-19 Pandemic: A Qualitative Study
Source: MDM Policy Pract. 2022 Jul 29;7(2):23814683221116362. doi: 10.1177/23814683221116362 (PMC9340948; doi:10.1177/23814683221116362)
Supplement: sj-docx-5-mpp-10.1177_23814683221116362 – Supplemental material for Use of Modeling to Inform Decision Making in North Carolina during the COVID-19 Pandemic: A Qualitative Study [file sj-docx-5-mpp-10.1177_23814683221116362.docx]

Appendix 5 -- Individual Organization Characteristics included in the Analysis

| **ID** | **Sector** | **Organization Type** | **Role in Organization** | **Region of NC** | **County Size** | **County Rurality** | **Size of Community/ Constituents** | **Race/Ethnicity of Community/ Constituents** | **Date of Interview** |
| --- | --- | --- | --- | --- | --- | --- | --- | --- | --- |
| Government 1 | Government | County Management | County Manager/ Attorney | Eastern | Urban population of 20,000 or more, adjacent to a metro area | Non-metropolitan | 25,000 - 50,000 citizens | Majority White | Feb 10, 2021 |
| Government 2 | Government | County Social Services | Director | Western | Completely rural or less than 2,500 urban population, not adjacent to a metro area | Non-metropolitan | 5000 - 10,000 citizens | Majority White | Nov 4, 2020 |
| Religious 1 | Religious | Church | Senior Pastor | Piedmont | Counties in metro areas of 1 million population or more | Metropolitan | active member count unknown | Majority White | Sept 22, 2020 |
| Education 1 | Education | University | Senior Vice Provost for Enrollment Management and Services | Piedmont | Counties in metro areas of 1 million population or more | Metropolitan | ~30,000 total students | Majority White, Minority Black and Asian | Oct 1, 2020 |
| Education 2* | Education | University | President | Eastern | Counties in metro areas of fewer than 250,000 population | Metropolitan | ~5,000 total students | Minority White and Black | Oct 14, 2020 |
| Education 3 | Education | County School Board | Member | Piedmont | Counties in metro areas of 250,000 to 1 million population | Metropolitan | ~7,000 total students | Majority White, Minority Black or Latino | Oct 28, 2020 |
| Education 4* | Education | County School Board | Superintendent | Eastern | Counties in metro areas of fewer than 250,000 population | Metropolitan | ~25,000 students | Minority White, Black, and Asian | Nov 4, 2020 |
| Education 5 | Education | County School Board | Member | Piedmont | Counties in metro areas of 1 million population or more | Metropolitan | ~150,000 students | Majority White, Minority Black and Latino | Nov 6, 2020 |
| Public Health 1 | Public Health | County Health Department | Director | Piedmont | Counties in metro areas of 250,000 to 1 million population | Metropolitan | ~300,000 citizens | Majority White, Minority Black | Sept 16, 2020 |
| Public Health 2 | Public Health | County Health Department | Director | Piedmont | Counties in metro areas of 1 million population or more | Metropolitan | >300,000 citizens | Majority White, Minority Black or Latino | Sept 21, 2020 |
| Public Health 3 | Public Health | County Health Department | Director | Piedmont | Counties in metro areas of 250,000 to 1 million population | Metropolitan | ~300,000 citizens | Majority White, Minority Black or Latino | Sept 25, 2020 |
| Public Health 4 | Public Health | County Health Department | Director | Piedmont | Counties in metro areas of 250,000 to 1 million population | Metropolitan | 100,000 - 150,000 citizens | Majority White, Minority Black or Latino | Sept 21, 2020 |
| Healthcare 1* | Healthcare | University | Director and Medical Director of Student Health Services | Piedmont | Counties in metro areas of 1 million population or more | Metropolitan | ~30,000 total students | Majority White, Minority Black and Asian | Sept 25, 2020 |
| Healthcare 2 | Healthcare | Statewide Healthcare Association | President | State-wide | State-wide | State-wide | State-Wide | State-wide Association | Sept 11, 2020 |
| Healthcare 3* | Healthcare | Private Health System | Systems Engineer | Eastern | Eastern Region | Multi | ~1,500 bed health system serving ~1M people in Eastern North Carolina | Majority White, Minority Black and Asian | Sept 18, 2020 |
| Business 1 | Business | Software Business | Director of Global Public Sector | State-wide | State-wide | State-wide | 10,000 - 15,000 employees | Unknown | Nov 3, 2020 |
| Public Safety 1 | Public Safety | County Emergency Services | Emergency Manager | Piedmont | Counties in metro areas of 250,000 to 1 million population | Metropolitan | >300,000 citizens | Majority White, Minority Black | Nov 10, 2020 |
| Public Safety 2 | Public Safety | University | Director, Emergency Management and Mission Continuity | Piedmont | Counties in metro areas of 1 million population or more | Metropolitan | ~30,000 total students | Majority White, Minority Black and Asian | Sept 21, 2020 |
| Public Safety 3 | Public Safety | County Emergency Services | Director | Piedmont | Counties in metro areas of 250,000 to 1 million population | Metropolitan | >300,000 citizens | Majority White, Minority Black | Nov 4, 2020 |

*Originally identified through personal connections of members of the research team.
